# Fibroblast diversity within human gut-associated lymphoid tissues

**DOI:** 10.1084/jem.20250471

**Published:** 2025-12-11

**Authors:** Urs M. Mörbe, Fredrik V. Junghus, Grigorii Nos, Peter B. Jørgensen, Melissa J. Ensmenger, Venla A. Väänänen, Mads D. Wewer, Gorm R. Madsen, Lene B. Riis, Henrik L. Jakobsen, Lars R. Olsen, Søren Brunak, Ole H. Nielsen, William W. Agace

**Affiliations:** 1 Department of Immunology & Microbiology, LEO Foundation Skin Immunology Research Center, University of Copenhagen, Copenhagen, Denmark; 2 Section for Translational and Experimental Immunology, Department of Health Technology, Technical University of Denmark, Kongens Lyngby, Denmark; 3 Novo Nordisk Foundation Center for Protein Research, Faculty of Health and Medical Sciences, University of Copenhagen, Copenhagen, Denmark; 4 Section for Bioinformatics, Department of Health Technology, Technical University of Denmark, Lyngby, Denmark; 5 Gastro Unit, Medical Section, Copenhagen University Hospital - Amager and Hvidovre, Hvidovre, Denmark; 6 Department of Pathology, Herlev Hospital, Copenhagen University Hospital, Herlev, Denmark; 7 Department of Surgery, Herlev Hospital, Copenhagen University Hospital, Herlev, Denmark; 8 Department of Public Health, Faculty of Health and Medical Sciences, University of Copenhagen, Copenhagen, Denmark; 9 Department of Gastroenterology, Herlev Hospital, University of Copenhagen, Herlev, Denmark; 10 Immunology Section, Lund University, Lund, Sweden

## Abstract

Gut-associated lymphoid tissues (GALT) represent major sites of adaptive immune priming in the intestine, yet our understanding of human GALT diversity and function remains limited. Here, we used single-cell RNA sequencing, flow cytometry, and confocal laser microscopy to map the fibroblast (FB) landscape of human GALT, including that of Peyer’s patches (PP), mucosal isolated lymphoid follicles (M-ILF), and submucosal ILF (SM-ILF). We identify CD24 as a marker that distinguishes GALT from other intestinal FB and demonstrate that CD24^+^ FB consist of distinct subsets that locate within discrete niches. We show that the composition and transcriptional profile of M-ILF and SM-ILF FB differs with SM-ILF FB appearing more focused at providing T cell support. Finally, we find the transcription profile of PP T zone reticular cells to be altered in Crohn’s disease and that cells with a GALT FB-like profile can be detected in other chronic inflammatory diseases. Collectively, our findings provide an important framework for understanding GALT diversity and function.

## Teaser

Specialized fibroblasts in secondary lymphoid tissues are essential for generating effective lymphocyte responses. Here, we characterize and spatially map the fibroblast landscape within human large and small intestinal gut-associated lymphoid tissues, including Peyer’s patches and isolated lymphoid follicles, during homeostasis and Crohn’s disease.

## Introduction

The intestinal mucosa is the major lining separating the luminal contents of the gut from the deeper sterile tissues. It consists of an epithelial monolayer and underlying lamina propria (LP), rich in immune cells essential for maintaining intestinal immune homeostasis. While the epithelium and LP are considered the key immune effector sites of the intestine, the mucosa also contains lymphoid structures, collectively termed gut-associated lymphoid tissues (GALT). GALT, together with intestinal draining mesenteric lymph nodes (MLN), represent the key adaptive immune priming sites of the intestine and include the multifollicular small intestinal Peyer’s patches (PP) and appendix, as well as isolated lymphoid follicles (ILF), found throughout the length of the intestine (Mörbe et al., 2021). Human GALT are highly organized, consisting of a marginal ring of naïve and memory T cells and a central cluster of B lymphocytes. They distinguish themselves from classical secondary lymphoid organs such as LN in containing a follicular-associated epithelium (FAE) rich in antigen sampling microfold (M cells) and an underlying antigen presenting cell-rich subepithelial dome (SED) (Mörbe et al., 2021; Spencer and Sollid, 2016; Spencer et al., 2009). Moreover, large GALT including PP and the appendix constitutively contain germinal centers (GC) (Reboldi and Cyster, 2016; Spencer and Sollid, 2016), presumably as a result of continual antigen stimulation from the intestinal lumen. Some differences exist in the anatomical location and cellular composition of human GALT: for example, during homeostasis most ILF within the large intestine penetrate the muscularis mucosae and locate within the underlying submucosa (SM), while ILF within the ileum locate almost exclusively within the mucosa (Fenton et al., 2020; Jørgensen et al., 2021). We recently termed these structures submucosal ILF (SM-ILF) and mucosal ILF (M-ILF) (Fenton et al., 2020; Jørgensen et al., 2021) to distinguish between them. The cellular composition of ileal M-ILF and colonic SM-ILF also differs with M-ILF containing a larger SED and glycoprotein-2–expressing M cells within their FAE and SM-ILF containing greater proportions of naïve CD4^+^ and CD8^+^ T cells (Fenton et al., 2020; Mörbe et al., 2021; Spencer et al., 2019). While the precise contributions of different GALT to intestinal immune responses remain to be clarified, the IgA repertoire of ileal PP most closely resembles that found in ileal LP, while that of colonic SM-ILF bears a closer resemblance to colonic LP (Fenton et al., 2020), suggesting they participate in the generation of regionalized adaptive immune responses.

Murine LN and PP contain distinct subsets of fibroblasts (FB) known as fibroblastic reticular cells (FRC) that collectively support the survival, migration, proliferation, and activation of lymphocytes (De Martin et al., 2024). B cell zone reticular cells (BRC), the most prominent of which are follicular dendritic cells (FDC), attract B cells (Legler et al., 1998) and T follicular helper cells (Kim et al., 2001) into GC by the secretion of migratory cues such as CXCL13 (Cyster et al., 2000; Mourcin et al., 2021), and aid the generation of plasma cells by presenting complement- and antibody-bound antigen (Fang et al., 1998; Roozendaal and Carroll, 2007). At the T cell zone border of the B cell follicle are additional CXCL13-producing BRC, also termed T/B border reticular cells (TBRC) (Lütge et al., 2023; Pikor et al., 2020). The SED of murine PP contains a specialized population of FB termed marginal reticular cells (MRC) that, through their expression of RANKL, play an essential role in stimulating M cell development (Knoop et al., 2009), and through their expression of CXCL13 presumably contribute to immune cell organization (Katakai et al., 2008). Lastly, T cell zone reticular cells (TRC), present in the T cell zone of LN and PP (Prados et al., 2021; Rodda et al., 2018), attract T cells and dendritic cells through the production of CCL19 and CCL21, produce IL-7 to promote T cell survival (Knop et al., 2020), and form a network that promotes immune cell migration and antigen dissemination (Acton et al., 2012; Gretz et al., 2000; Krishnamurty and Turley, 2020; Link et al., 2011). While recent studies have started to assess FB diversity in human lymphoid tissues such as the tonsils (De Martin et al., 2023; Mourcin et al., 2021), and FDC have been observed in human GALT (Yamanaka et al., 2001), the extent of FB diversity within human GALT and whether this differs between GALT structures remain unclear. Such studies are important given the profound differences in GALT distribution, composition, and development between humans and mice (Mörbe et al., 2021).

Here, we used single-cell RNA sequencing (scRNA-seq), confocal laser microscopy, and flow cytometry to comprehensively map the FB landscape of human GALT. We identified six transcriptionally distinct and spatially restricted FB subsets that were present during homeostasis and in Crohn’s disease (CD). We also described the differences between ileal and large intestinal GALT and set our findings into the context of previously published studies, finding that cells with a GALT FRC-like phenotype are present in other chronic inflammatory diseases.

## Results

### The human intestine contains four major transcriptionally distinct FB subsets

**Figure 1.**
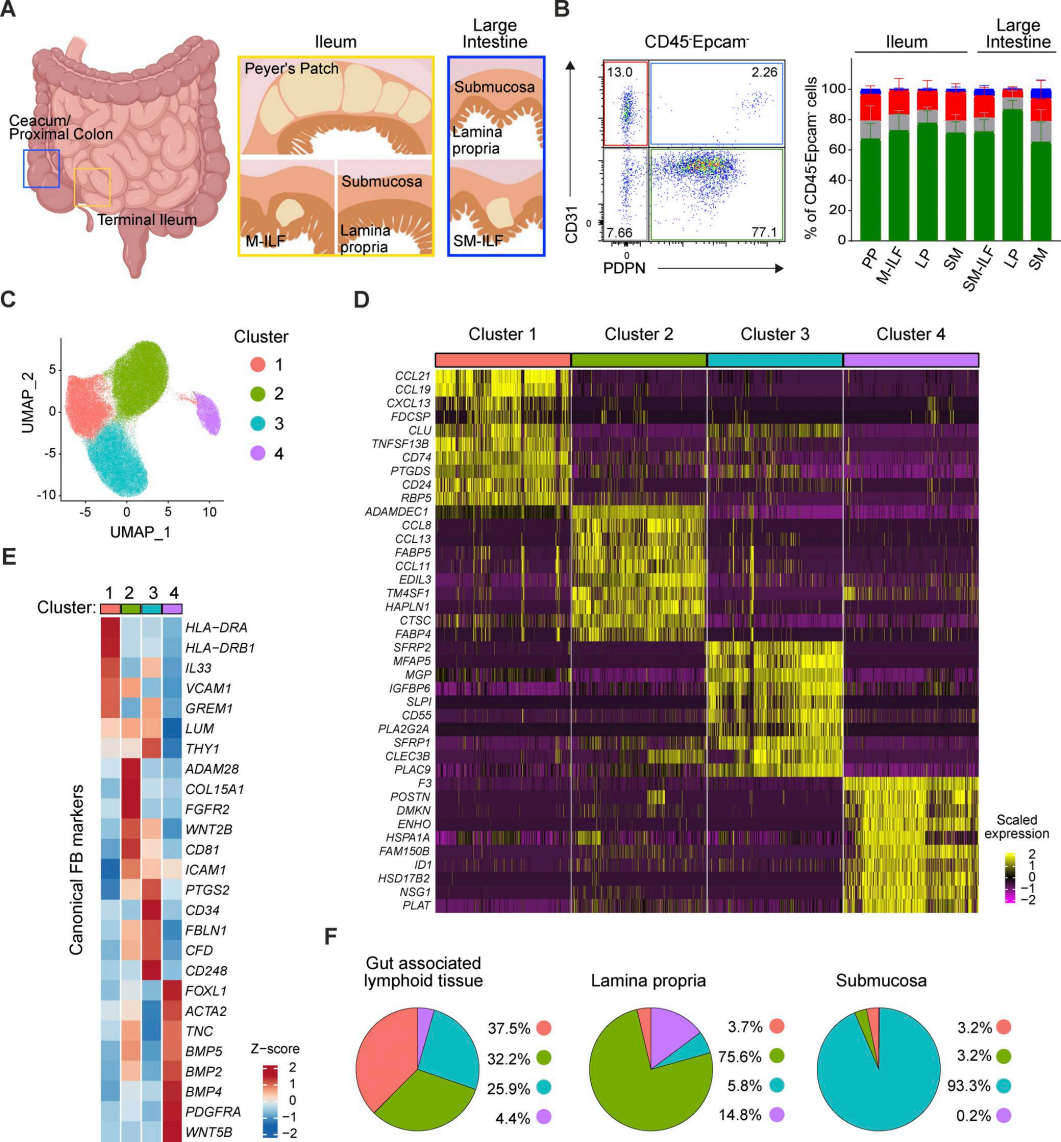
**scRNA-seq reveals four distinct intestinal FB clusters. (A)** Schematic overview showing the anatomic sites of the human intestine from which samples were obtained. **(B)** Left panel: Representative stromal cell gate showing CD31^+^PDPN^−^ vascular endothelial cells (red), CD31^+^PDPN^+^ lymphatic endothelial cells (blue), CD31^−^PDPN^+^ FB (green), and the CD31^−^PDPN^−^ double negative cells (gray). Right panel: Quantification of the stromal cell fractions in the indicated isolated intestinal layers. The plot shows the means ± SD of 20 (large intestinal LP and SM-ILF), 17 (ileal LP and PP), 10 (large intestinal SM), 6 (ileal SM), or 4 (ileal M-ILF) tissue donors; pooled data are shown from 19 independent experiments. **(C)** UMAP representation of unsupervised Louvain clustering of 101,401 intestinal FB pooled from the LP, SM, and GALT. **(D)** Heatmap showing the top ten DEG for the four identified FB clusters. **(E)** Heatmap showing expression levels of canonical FB markers by the four identified intestinal clusters. **(F)** Pie chart representation of FB cluster abundance in the scRNA-seq data object at the indicated sites. Results in C–F are pooled from five (PP and SM-ILF), three (ileal and large intestinal LP and M-ILF), and one (ileal and large intestinal SM) tissue donors.

**Figure S1.**
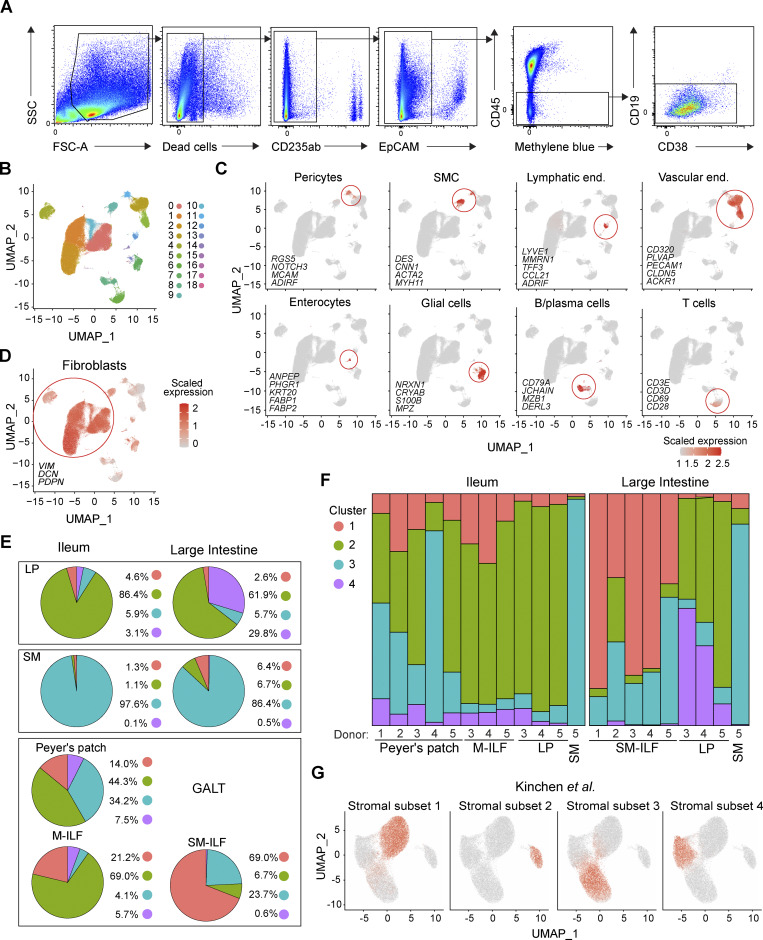
**Generation, processing, and analysis of scRNA-seq FB datasets. (A)** Representative flow cytometry plots showing the gating strategy for the identification and sorting of stromal cells, excluding dead cells, CD235ab^+^ red blood cells, EpCAM^+^ epithelial cells, CD45^+^ immune cells, and CD45^lo^CD19^+/−^CD38^hi^ plasma cells. **(B)** UMAP representation of scRNA-seq results before removal of contaminating non-FB. **(C)** Identification of contaminating non-FB based on gene sets characteristic for the indicated cell types. **(D)** Identification of FB based on their expression of the indicated FB signature markers. Red circle marks FB. **(E)** Pie charts showing the abundance of FB clusters pooled by tissue and site of origin. **(F)** Bar plot showing the abundance of each FB cluster as a fraction of total FB in each sample by donor and tissue origin. **(G)** Identification of intestinal FB subsets according to Kinchen et al. (2018) on the combined dataset including all LP, SM, and GALT-derived cells based on an overlay of the top 10 DEG per cluster identified in the original Kinchen et al. (2018) publication. **(B–G)** Data include cells from five tissue donors (5× PP and SM-ILF, 3× M-ILF and LP, 1× SM). All available tissues from a given donor were processed together in a single experiment, and each donor was processed separately.

To assess FB diversity within and along the human intestine, human ileal and large intestinal GALT (PP, M-ILF, and SM-ILF), LP, and SM were isolated from surgical resections of patients with colorectal cancer (CRC) >10 cm from the tumor site as previously described (Fenton et al., 2020; Jørgensen et al., 2021) (Fig. 1 A). Following tissue digestion, podoplanin (PDPN)^+^CD31^+^ lymphatic endothelial cells, PDPN^−^CD31^+^ vascular endothelial cells, CD31^−^PDPN^−^ double negative cells, and PDPN^+^CD31^−^ FB were readily identified in cell suspensions from all tissues by flow cytometry (Fig. 1 B; for pregating, see Fig. S1 A). To gain a greater understanding of FB diversity within these distinct compartments, we performed scRNA-seq on freshly isolated flow cytometry–sorted EpCAM^−^CD235ab^−^CD45^−^ cells from ileal LP (3×), PP (5×), M-ILF (3×), and SM (1×), as well as large intestinal LP (3×), SM-ILF (5×), and SM (1×) from five individual donors (see Table S1 for donor information). After data integration, cell doublets, and dying and contaminating cells were identified and bioinformatically removed from the dataset (Fig. S1, B and C), with remaining cells expressing known FB signature genes (Elmentaite et al., 2021; Kinchen et al., 2018) (Fig. S1 D). Batch correction, unsupervised Louvain clustering, and Uniform Manifold Approximation and Projection (UMAP) dimensionality reduction on the remaining 101,401 FB identified four major FB clusters (Fig. 1 C). The analysis of top differentially expressed genes (DEG) and canonical FB markers (Brügger and Basler, 2023) revealed marked differences between each of these clusters (Fig. 1 D and Table S2), suggesting that they represented functionally distinct FB subsets. Cluster 1 expressed transcripts encoding for the chemokines *CCL19*, *CCL21*, and *CXCL13*, the secreted chaperone clusterin (*CLU*), and *CD74*, the major histocompatibility complex II (MHCII) invariant chain, all genes previously associated with human (De Martin et al., 2023; Mourcin et al., 2021; Verbrugghe et al., 2008) and murine (Cheng et al., 2019, 2022; Prados et al., 2021; Rodda et al., 2018) FRC, as well as putative FRC-like FB in the human intestine (Kinchen et al., 2018). Cluster 1 also expressed the MHCII genes *HLA-DRA* and *HLA-DRB1* as top DEG (Fig. 1 E and Table S2), similar to FRC in murine LN that also express MHCII (Baptista et al., 2014; Nadafi et al., 2020; Shaikh et al., 2022). Cluster 2 expressed transcripts encoding the matrix metalloprotease *ADAMDEC1* and the chemokines *CCL8*, *CCL11*, and *CCL13* (Fig. 1, D and E; and Table S2), known markers of LP FB (Kinchen et al., 2018; Martin et al., 2019). Cluster 3 expressed *CD34*, *PTGS2*, and *PI16* (Fig. 1, D and E; and Table S2), markers previously associated with adventitial-type FB (Buechler et al., 2021; Gong et al., 2022; Korsunsky et al., 2022; Sitnik et al., 2016), and cluster 4 expressed the cell surface glycoprotein *F3* (tissue factor) and genes associated with the Wnt/BMP signaling pathway (Fig. 1, D and E; and Table S2), previously associated with subepithelial FB (SE-FB) (Kinchen et al., 2018; Smillie et al., 2019). Congruent with these findings, the abundance of each cluster differed between intestinal compartments with cluster 1 enriched in GALT, cluster 2 enriched in LP, cluster 3 enriched in SM, and cluster 4 enriched in LP and ileal GALT (Fig. 1 F; and Fig. S1, E and F). Mapping these clusters to intestinal FB subsets described in Kinchen et al. indicated that cluster 1 was most similar to the FRC-like proinflammatory “stromal subset 4” in Kinchen et al., cluster 2 to the LP bulk FB “stromal subset 1,” cluster 3 to “stromal subset 3” rich in genes for extracellular matrix organization, and cluster 4 to the colonic crypt niche “stromal subset 2” (Kinchen et al., 2018) (Fig. S1 G).

### GALT-associated FRC express CD24

**Figure 2.**
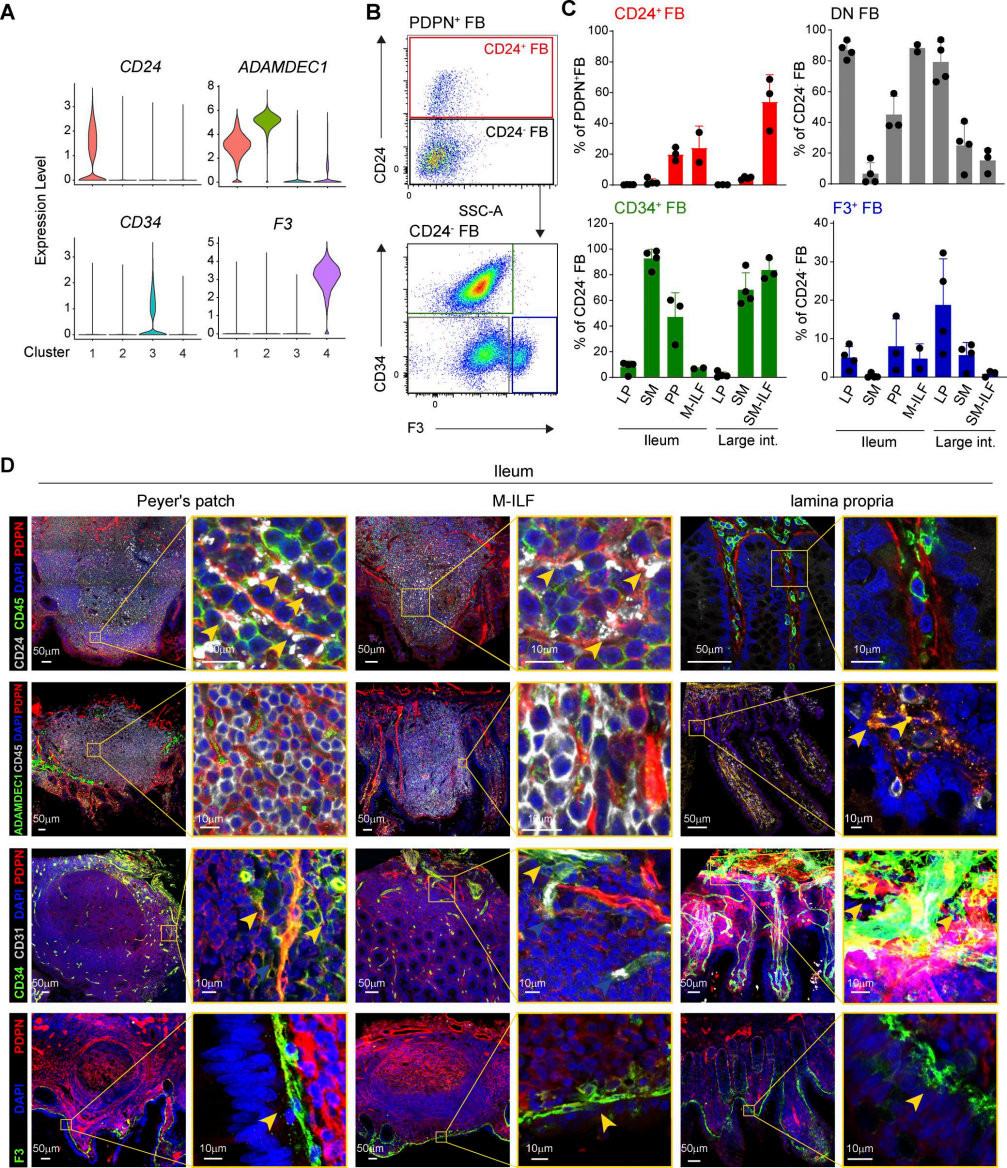
**Flow cytometry and histological analyses of intestinal FB confirm heterogeneity between sites. (A)** Violin plots showing the expression of the indicated genes by intestinal FB pooled from five (PP and SM-ILF), three (ileal and large intestinal LP and M-ILF), and one (ileal and large intestinal SM) samples. **(B)** Representative flow cytometry plots showing the gating of CD24^+^ FB within the total pool of PDPN^+^ FB and the gating of CD34^+^ and F3^+^ FB among CD24^−^ FB. Cells were pregated on live, CD235ab^−^EpCAM^−^CD45^−^CD31^−^PDPN^+^ cells. **(C)** Mean abundance (±SD) of CD24^+^ FB among total PDPN^+^ FB (upper left) and CD34^−^F3^−^ DN (upper right), CD34^+^ FB (lower left), and F3^+^ FB (lower right) among CD24^−^ FB. Each symbol represents one sample of two (M-ILF), three (PP and SM-ILF), or four (ileal and large intestinal SM and LP) tissue donors. Pooled data are from four separate experiments. **(D)** Confocal laser microscopy images of ileal GALT and surrounding LP. Yellow boxes in left panels highlight magnified areas. PDPN was chosen to highlight all FB, CD24, ADAMDEC1, CD34, and F3, the indicated FB subsets, and CD31 to mark endothelial cells. Yellow arrows mark ADAMDEC1^+^PDPN^+^, CD34^+^PDPN^+^, and F3^+^PDPN^+^ double positive FB, respectively. Images are representative of ≥3 replicates from different donors per staining that were stained in three or four independent experiments. DN, double negative.

**Figure S2.**
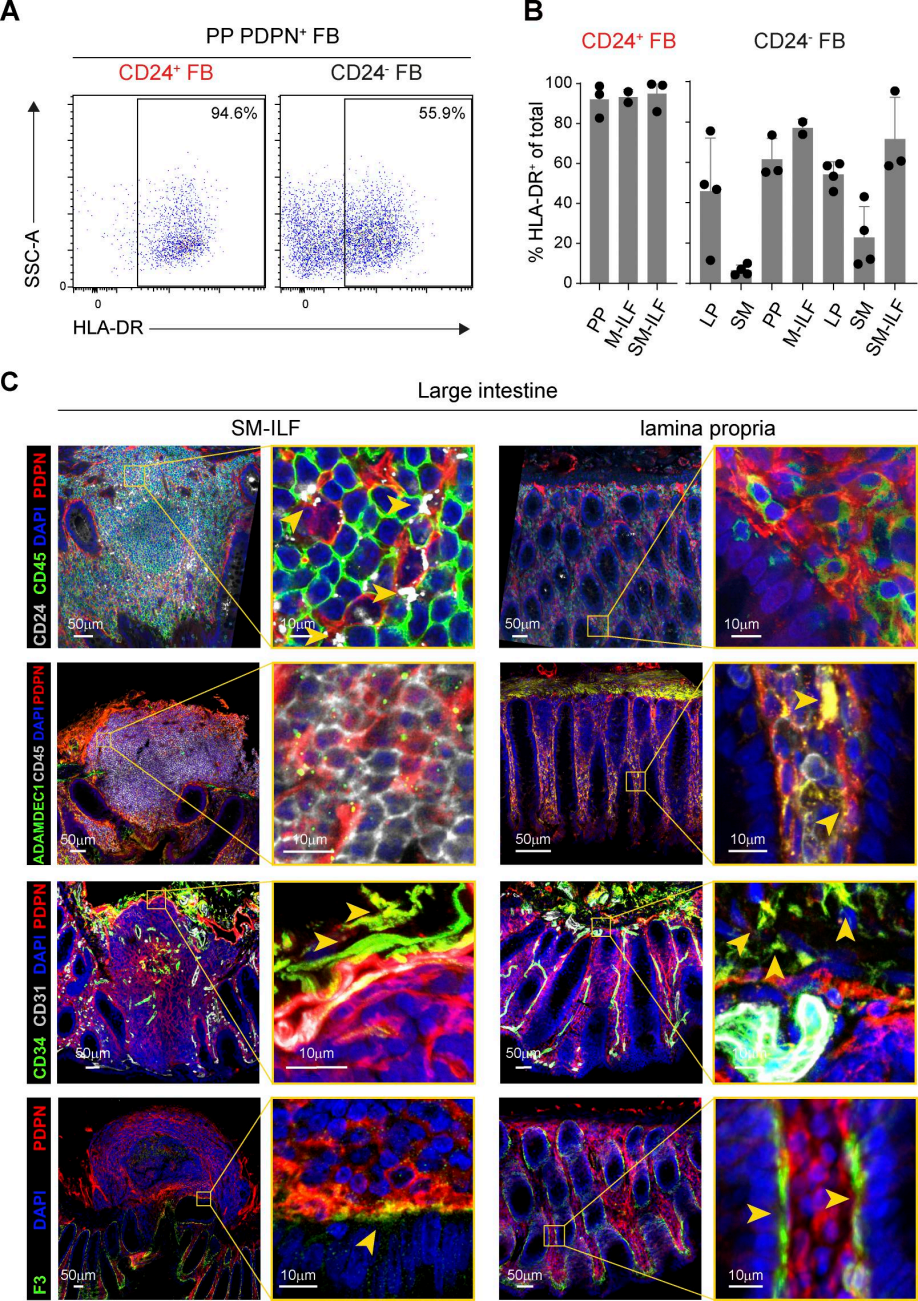
**Validation of scRNA-seq results using flow cytometry and confocal laser microscopy. (A)** Representative flow cytometry plots showing the expression of HLA-DR on PP-derived CD24^+^PDPN^+^ FB (left panel) and CD24^−^PDPN^+^ FB (right panel). **(B)** Quantification of HLA-DR^hi^ cells among CD24^+^PDPN^+^ FB (left panel) and CD24^−^PDPN^+^ FB (right panel). Dots represent individual samples; bars show the mean of data pooled from two (M-ILF), three (PP and SM-ILF), or four (SM, LP) tissue donors, and whiskers indicate SD. Pooled data are from four separate experiments. **(C)** Confocal laser microscopy images of large intestinal SM-ILF (left) and surrounding LP (right). Yellow boxes in the left panels highlight magnified areas. PDPN was chosen to highlight all FB, CD24, ADAMDEC1, CD34, and F3, the indicated FB subsets, and CD31 was chosen to highlight endothelial cells. The yellow arrows mark ADAMDEC1^+^PDPN^+^, CD34^+^PDPN^+^, and F3^+^PDPN^+^ FB, respectively. DAPI highlights all nucleated cells. Results are from one representative image of ≥3 replicates from different donors per staining that were stained in independent experiments.

To validate our scRNA-seq findings and determine where the four FB subsets were located within the intestinal wall, we analyzed our scRNA-seq datasets for DEG of potential use in identifying each FB subset by flow cytometry and immunohistochemistry. *CD24* (heat stable antigen) was identified as a marker for FB cluster 1, matrix metalloprotease-encoding gene *ADAMDEC1* for FB cluster 2, *CD34* for FB cluster 3, and *F3* for FB cluster 4 (Fig. 2 A). Consistent with our scRNA-seq analysis, flow cytometry analysis of PDPN^+^ FB identified a population of CD24^+^ FB that were enriched in GALT cell suspensions and a population of CD24^−^F3^−^CD34^+^ FB that were enriched in SM cell suspensions, as well as PP and SM-ILF (Fig. 2, B and C), both of which extend into the SM (Jørgensen et al., 2021). Also consistent with our scRNA-seq data, we found that the majority of CD24^+^ FB expressed HLA-DR, while CD24^−^ FB did this to a lower extent (Fig. S2, A and B), compatible with findings that FRC in murine LN express high levels of MHCII (Baptista et al., 2014; Nadafi et al., 2020; Shaikh et al., 2022). Flow cytometry analysis also identified a population of CD24^−^CD34^−^F3^+^ FB that were enriched in LP samples and were also present in ileal M-ILF that sit within the mucosa, as well as PP (Fig. 2 C). A small, but variable, fraction of CD24^−^CD34^−^F3^+^ FB was also detected in colonic SM cell suspensions (Fig. 2 C), and we hypothesize that these cells are contaminants from the colonic LP, which contained the largest fraction of CD24^−^CD34^−^F3^+^ FB (Fig. 2 C). Finally, we also confirmed scRNA-seq findings showing that PDPN^+^ FB lacking expression of CD24, CD34, and F3, likely representing cluster 2 FB, were enriched in LP samples and ileal M-ILF (Fig. 2 C).

Having identified putative markers that distinguish each of the four FB subsets, we performed immunohistochemical analysis to determine their location within the intestinal wall. PDPN^+^CD24^+^ FB were selectively located throughout ileal and large intestinal GALT but were absent from surrounding LP or SM (Fig. 2 D and Fig. S2 C), indicating that CD24 is selectively expressed on GALT FRC. ADAMDEC1 was expressed at high levels by interstitial FB (Int-FB) in the ileum and colon LP, and weakly by GALT FRC and by a line of FB at the basolateral side of the mucosa (Fig. 2 D and Fig. S2 C), the latter most likely representing smooth muscle cells of the muscularis mucosa (Muhl et al., 2022). PDPN^+^CD34^hi^ FB were primarily located in the SM on the basolateral side of GALT and LP (Fig. 2 D and Fig. S2 C), while CD34^+^ cells in GALT and LP parenchyma expressed CD31 but not PDPN (Fig. 2 D and Fig. S2 C) and were thus endothelial cells (Vannucchi et al., 2013). Consistent with previous findings (Kinchen et al., 2018), PDPN^+^F3^hi^ FB were located as a thin layer directly underlying the epithelium in both GALT and LP and thus represent SE-FB (Fig. 2 D and Fig. S2 C). Taken together, these results demonstrate that human intestinal GALT, LP, and SM contain transcriptionally distinct FB subsets and that CD24 can be used as a marker to identify GALT FRC (cluster 1).

### Human PP and M-ILF contain transcriptionally distinct subsets of *CD24*^+^ FRC that occupy distinct GALT niches

**Figure S3.**
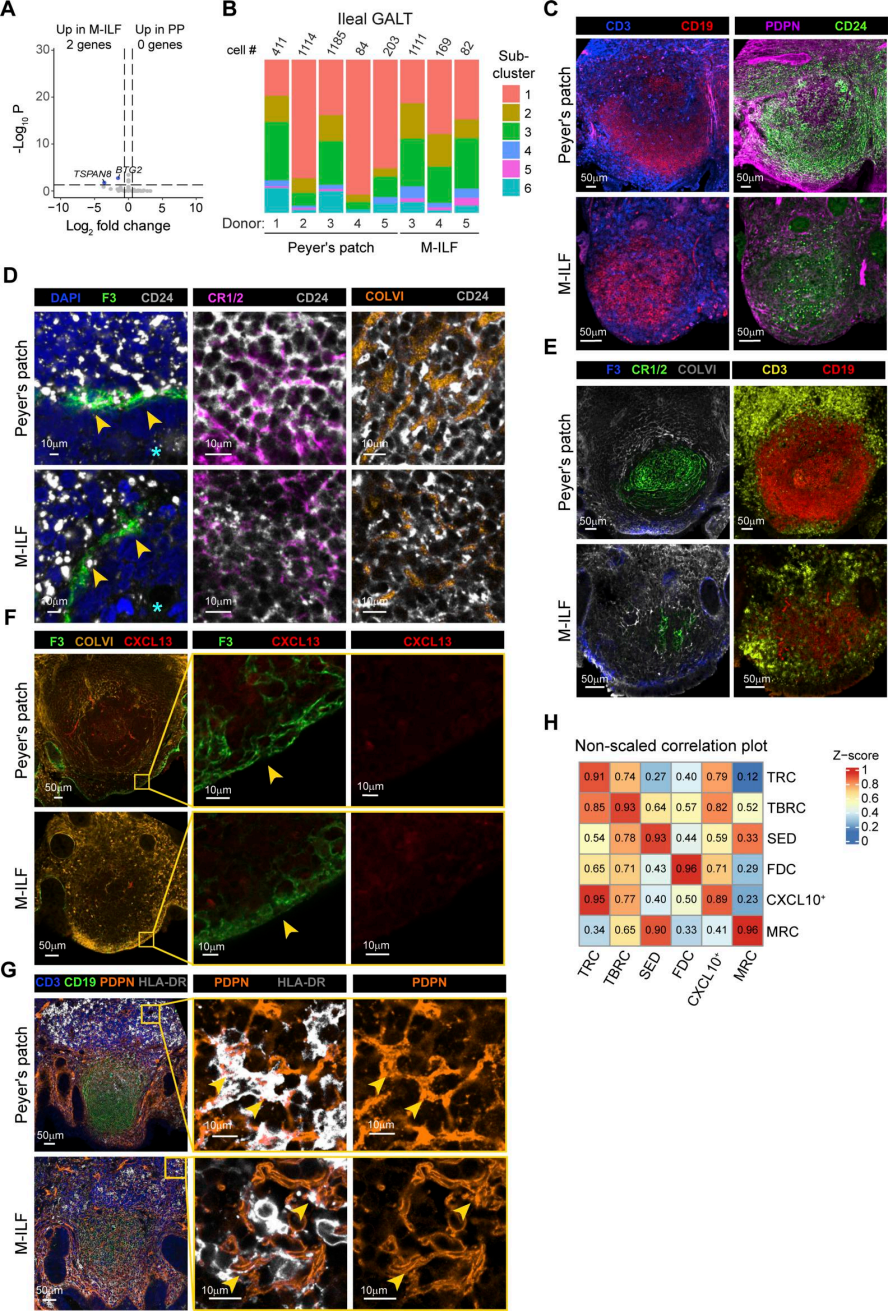
**Distribution of GALT** **FRC** **subsets in ileal GALT. (A)** Volcano plot showing DEG between GALT FRC from PP and M-ILF. **(B)** Bar plot showing FRC subset abundance among GALT FRC in the individual ileal GALT samples. **(C)** Confocal laser microscopy images showing CD3^+^ T cells, CD19^+^ B cells (left panels), and CD24-expressing FB among all PDPN^+^ FB (right panels) in ileal PP (upper panels) and M-ILF (lower panels). **(D)** Confocal laser microscopy images showing the expression of CD24 on F3^+^/CR1/2^+^/COLVI^+^ FB within ileal PP (upper panels) and M-ILF (lower panels). Star marks the apical side of the epithelial layer facing the intestinal lumen. **(E)** Confocal laser microscopy images showing the distribution of CD3^+^ T cells, CD19^+^ B cells, and F3^+^/CR1/2^+^/COLVI^+^ FB. **(F)** Confocal laser microscopy images showing the expression of CXCL13 levels by FB in the SED region. **(G)** Confocal laser microscopy images showing the expression of HLA-DR in ileal GALT. Images in C–G show one representative GALT out of replicates from two or three patients that were stained in independent experiments. **(H)** Nonscaled comparison of expression profiles of the indicated GALT FRC subclusters in ileal PP (y-axis) and M-ILF (x-axis). Zero (blue) indicates no overlap of the gene signatures; one (red) indicates full overlap. (A, B, and H) Data are from five independently processed tissue donors (5× PP and SM-ILF, 3× M-ILF).

**Figure 3.**
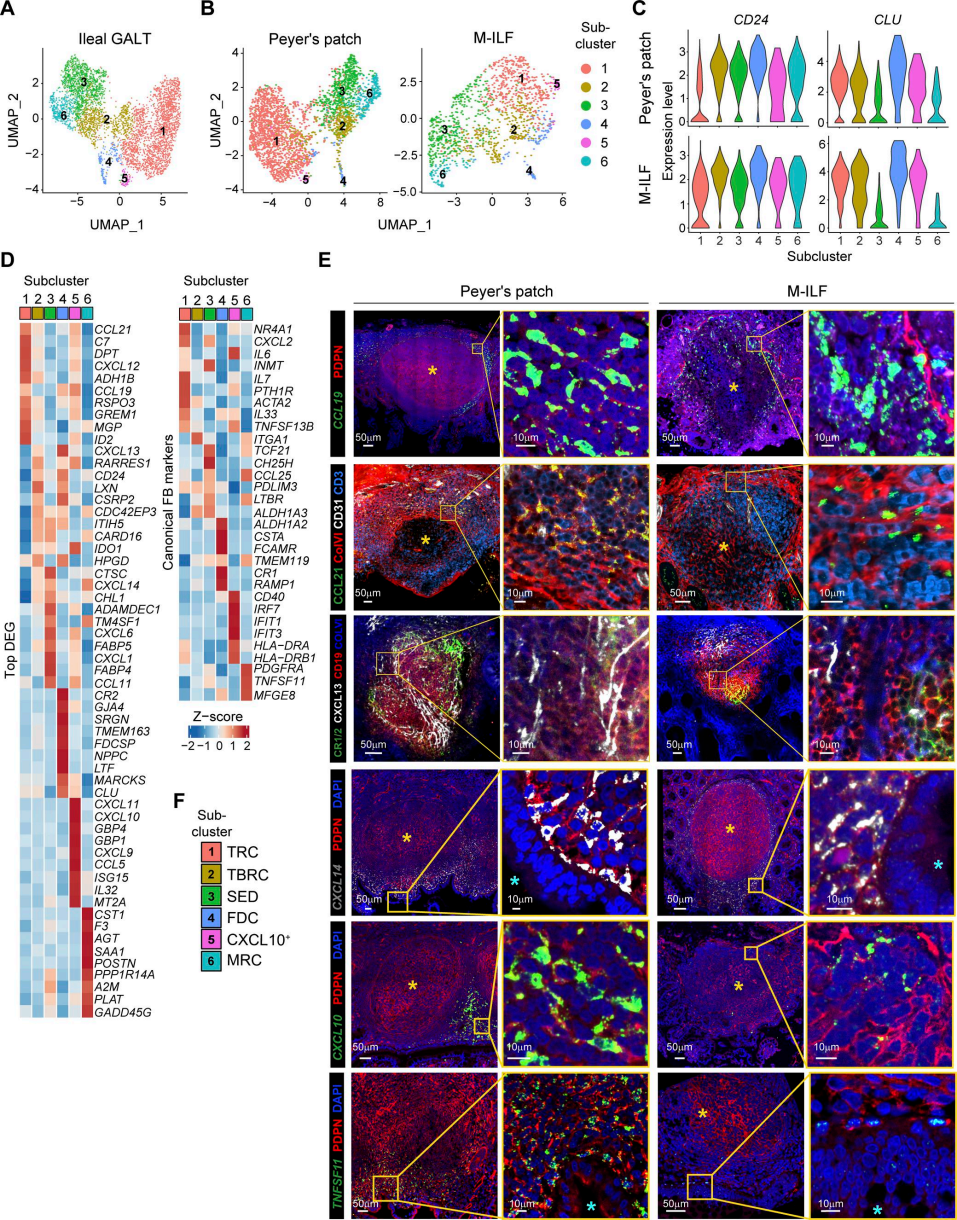
**FRC** **in human ileal GALT include spatially distinct subclusters. (A)** UMAP representation of pooled ileal GALT FRC showing the six subclusters. **(B)** UMAP representations of GALT FRC only from PP (left plot) and M-ILF (right plot). **(C)** Violin plots showing the expression levels of the FRC markers *CD24* and *CLU* by the indicated ileal GALT FRC subclusters of PP (upper panels) and M-ILF (lower panels). **(D)** Heatmaps showing the scaled expression of the top ten DEG (left panel) and other canonical FRC markers (right panel) by the indicated subclusters. **(A–D)** Data based on 4,359 single cells from five (PP, 2,997 cells) and three (M-ILF, 1,362 cells) independently processed donors. **(E)** Confocal laser microscopy images of ileal GALT highlighting the expression of the indicated stromal and immune cell markers either at the protein (PDPN, CCL21, CD31, ColVI, CD3, CR1/2, CXCL13, CD19) or at the RNA (*CCL19*, *CXCL14*, *CXCL10*) level. The cyan star marks the apical side of the epithelial layer facing the intestinal lumen; the yellow star marks the follicle center. Images are representative of three replicates derived from different patients that were stained in two or three independent experiments. **(F)** Cluster identity key linking numerical FRC clusters with FRC subcluster designations. *CXCL9*^*+*^*CXCL10*^*+*^ FRC are abbreviated as CXCL10^+^ FRC in this and subsequent figures.

Recent studies have suggested that murine small intestinal ILF contain a homogeneous FB population expressing *Cxcl13*, *Ccl19*, and *Clu* without spatially segregated subsets (Cheng et al., 2022; Knoop et al., 2011; Tsuji et al., 2008). To gain a deeper understanding of human GALT FRC diversity, *CD24*^+^ GALT FRC were bioinformatically isolated from PP and M-ILF datasets (scRNA-seq data from 4,359 cells) and further analyzed. Pseudobulk analysis showed that FRC of PP and M-ILF appeared transcriptionally like one another with few identified DEG (Fig. S3 A), and we thus fused the FRC datasets from PP and M-ILF (ileal GALT). Subclustering of ileal GALT FRC revealed six subclusters that were present in all M-ILF and PP samples albeit in varying proportions (Fig. 3 A, Fig. S3 B, and Table S3). To ensure that dataset integration had not obscured biological differences between M-ILF and PP, we also performed clustering on the M-ILF and PP datasets separately. This analysis confirmed the presence of all six clusters in both tissues (Fig. 3 B and Table S3), and we found that all FRC subclusters expressed *CD24* and *CLU* (Fig. 3 C). To gain insights into the identity and putative functions of these clusters, we assessed the top DEG and expression of canonical FB markers, with a focus on FRC markers (Ding et al., 2020; Mourcin et al., 2021; Pærregaard et al., 2023; Prados et al., 2021; Rodda et al., 2018), for each cluster (Fig. 3 D and Table S3). FRC subcluster 1 expressed high levels of the T cell chemoattractants *CCL19*, *CCL21*, and the T cell survival factor *IL7* and the cytokine *IL6* (Fig. 3 D), reminiscent of TRC present in murine secondary lymphoid tissues (Brown et al., 2019; Kapoor et al., 2021; Pezoldt et al., 2018; Prados et al., 2021; Rodda et al., 2018) and human tonsils (De Martin et al., 2023; Mourcin et al., 2021). Subcluster 2 expressed the B cell chemoattractant *CXCL13*, but not the complement receptors *CR1*/*CR2* indicative for FDC, and expressed lower levels of *CCL19* and *CCL21* (Fig. 3 D), suggesting that these cells represented TBRC (Lütge et al., 2023; Pikor et al., 2020). Subcluster 3 expressed the highest levels of the myeloid chemoattractant *CXCL14* (Fig. 3 D), previously associated with a subepithelial location in mice (Muhl et al., 2020) and humans (Elmentaite et al., 2021; Kinchen et al., 2018), *TCF21* (Fig. 3 D), a marker of SED-FB in mice (Ding et al., 2020), and the oxysterol synthesizing enzyme *CH25H* (Fig. 3 D), produced by FRC in the outer follicle and interfollicular areas of murine secondary lymphoid tissues (Rodda et al., 2018). Subcluster 4 expressed the highest levels of *CXCL13* and the FDC-associated genes *CR2* and *FDCSP* (Fig. 3 D), while subcluster 5 expressed interferon-stimulated genes including *GBP4*, *GBP5* (Tretina et al., 2019), *CXCL9*, and *CXCL10* (Tokunaga et al., 2018) (Fig. 3 D), and in this respect resembled the interferon-stimulated or virally infected FB identified in murine LN (Perez-Shibayama et al., 2020; Rodda et al., 2018). Finally, subcluster 6, while expressing the GALT FRC-associated markers *CD24* and *CLU* (Fig. 3 C), also expressed high levels of the SE-FB–associated markers *F3, AGT*, and *POSTN* (Fig. 1, D and E; Fig. 3 D; and Table S2 [Kinchen et al., 2018]), as well as the MRC-associated marker *TNFSF11* (Fig. 3 D) (Nagashima et al., 2017; Prados et al., 2021).

A key function of FRC is that they form spatially segregated niches to optimally support T and B cell survival and activation (De Martin et al., 2024). To determine whether the identified ileal GALT FRC subsets localized within spatially distinct niches, we performed immunohistochemical and RNAscope-based analyses of human PP and M-ILF (Fig. 3 E and Fig. S3, C–G). CD24^+^PDPN^+^ FRC were detected in both peripheral T cell zones and central B cell zones of ileal GALT (Fig. S3 C). In murine PP, FRC subsets include CR1/2^+^ FDC in the follicle center, ColVI^+^ TRC/TBRC in peripheral regions, and MRC at the SED (Prados et al., 2021). Similarly, human GALT contained CR1/2^+^ FRC in B cell–rich centers, ColVI^+^ FRC at T cell–rich margins, and some F3^+^ FRC in the SED, all of which expressed CD24 (Fig. S3, D and E), indicating conservation of these core FRC subsets across species.

We next sought to validate our scRNA-seq data and spatially localize these FRC subsets by selecting markers based on subset-defining DEG, as well as established FRC and immune cell markers. *CCL19*-expressing PDPN^+^ FB were readily detected at the follicle margins of GALT (Fig. 3 E). While we found it technically not possible to stain for CCL21 together with CCL19, PDPN, and CD3, CCL21 colocalized with the TRC marker ColVI (ER-TR7) in immediate proximity to CD3^+^ T cells within the marginal T cell zones (Fig. 3 E), suggesting that these cells likely represent TRC (Katakai et al., 2004; Prados et al., 2021). Staining for CXCL13, ColVI, CR1/CR2, and CD19^+^ B cells confirmed that ileal GALT contained CXCL13^+^ FDC and CXCL13^+^ non-FDC (Fig. 3 E), likely representing other BRC such as TBRC (Lütge et al., 2023; Pikor et al., 2020). In line with our scRNA-seq findings (Fig. 3 D), but in contrast to murine PP and LN (Katakai et al., 2008; Prados et al., 2021), as well as human LN (Lütge et al., 2023), CXCL13 staining was low on F3^+^ FRC within the SED (Fig. S3 F), indicating a more restricted expression of CXCL13 in human GALT. *CXCL14*^*hi*^ FRC were mostly detected within the SED area and to some extent within the interfollicular areas of PP (Fig. 3 E). *CXCL10*^+^ FRC appeared more in the follicle periphery (Fig. 3 E), pointing toward a primarily T cell zone location of these cells. Although it was technically not feasible to directly stain CXCL10 together with FB, T, and B cell markers, we were able to stain CD3^+^ T cells and CD19^+^ B cells in combination with the FB marker PDPN and the MHCII cell surface molecule HLA-DR, which was among the top DEG in the *CXCL9*^*+*^*CXCL10*^+^ FRC cluster (Fig. 3 D). HLA-DR was expressed by PDPN^-^ cells that had a dendritic cell-like morphology and on a subset of PDPN^+^ FB located adjacent to regions rich in CD3^+^ T cells (Fig. S3 G), underpinning that this cluster resides at the peripheral T cell zones of ileal GALT. *TNFSF11*-expressing FB were present in the SED region (Fig. 3 E), as previously observed in murine PP (Nagashima et al., 2017; Prados et al., 2021). In line with our scRNA-seq results showing that *TNFSF11*^+^ subcluster 6 also expressed high levels of *CXCL14* (Fig. 3 D), *TNFSF11*-expressing cells in the SED spatially overlapped with *CXCL14*^+^ cells, as the probe against *CXCL14* also marked cells directly below the epithelial layer of the SED (Fig. 3 E).

Considering our scRNA-seq and immunohistochemical analyses, we identified the *CCL19*^*+*^ FRC subcluster 1 as TRC, the *CXCL13*^*+*^*CR1*^−^*/2*^−^ FRC subcluster 2 as TBRC, the *CXCL14*^*+*^ FRC subcluster 3 as SED-FRC, the *CXCL13*^*+*^*CR1*^*+*^*/2*^*+*^ FRC subcluster 4 as FDC, the *CXCL9*^*+*^*CXCL10*^+^ FRC subcluster 5 as *CXCL9*^*+*^*CXCL10*^+^ FRC, and the *F3*^+^*TNFSF11*^+^ FRC subcluster 6 as MRC (Fig. 3 F).

### The FRC landscape of SM-ILF is partially distinct from that of ileal GALT

**Figure 4.**
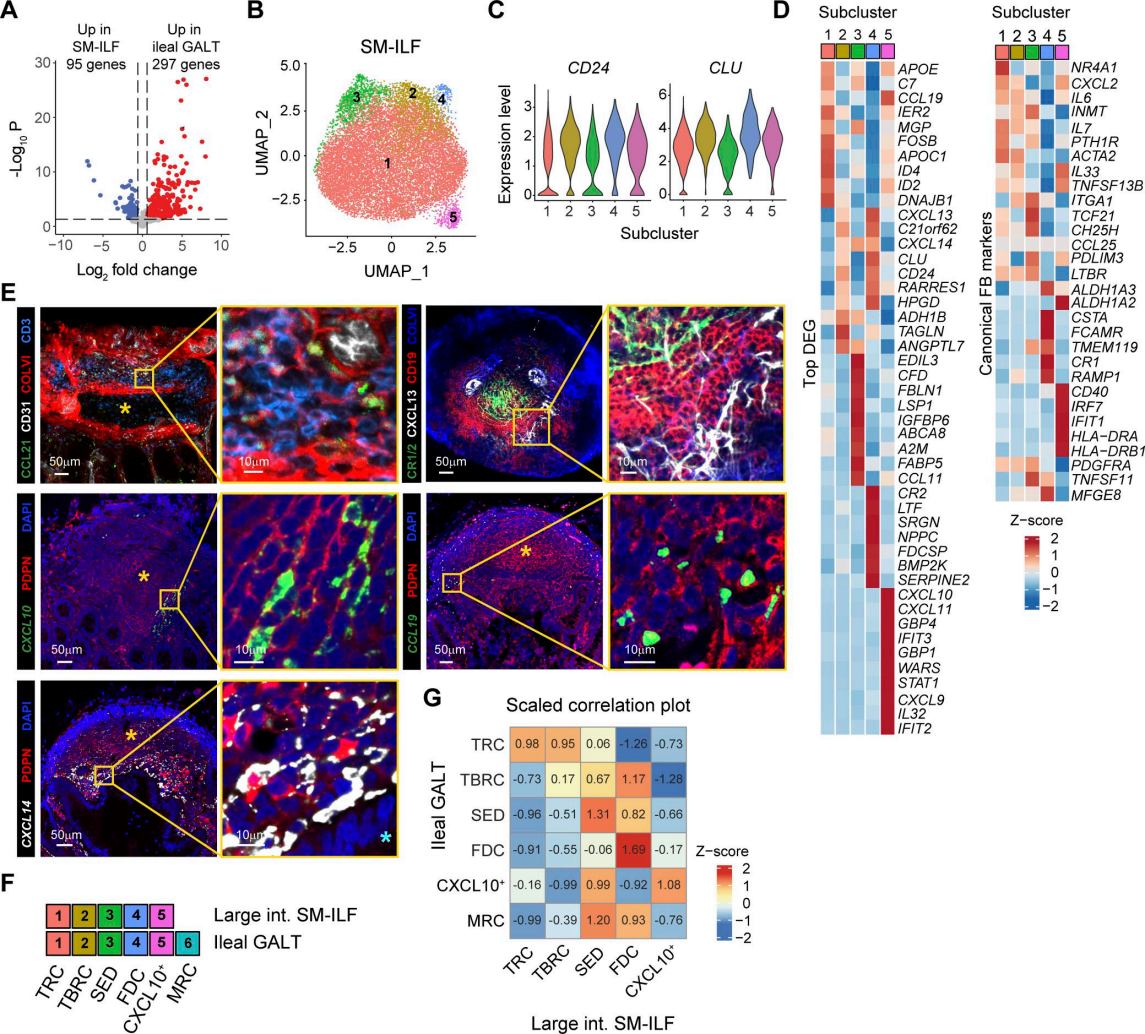
**FRC landscape of large intestinal SM-ILF. (A)** Volcano plot showing DEG between GALT FRC from large intestinal SM-ILF and ileal GALT based on data from five independently processed donors (5× PP and SM-ILF, 3× M-ILF). **(B)** UMAP representation of large intestinal SM-ILF GALT FRC showing five subclusters. **(C)** Violin plots showing expression of the FRC markers *CD24* and *CLU* by the indicated SM-ILF FRC subclusters. **(D)** Heatmaps showing the scaled expression of the top 10 DEG (left panel) and other canonical FB markers (right panel) by the indicated SM-ILF GALT FRC subclusters. **(B–D)** Data based on 14,786 single cells from five independently processed donors. **(E)** Confocal laser microscopy images of large intestinal SM-ILF showing expression of the indicated stromal and immune cell markers either at the protein (PDPN, CCL21, CD31, ColVI, CD3, CR1/2, CXCL13, CD19) or at the RNA (*CCL19*, *CXCL14*, *CXCL10*) level. The cyan star marks the apical side of the epithelial layer facing the intestinal lumen; the yellow star marks the follicle center. Images are representative of three replicates derived from different patients that were stained in two or three independent experiments. **(F)** Cluster identity key linking numerical FRC clusters with FRC subcluster designations in large intestinal (large int.) SM-ILF and ileal GALT. **(G)** Pearson correlation plots showing the overlap of FB subcluster signatures between ileal and large intestinal GALT FRC, based on all variable genes per cluster. Data are scaled within the same row, with the mean expression level of a signature score across all clusters in a row being set to zero.

**Figure S4.**
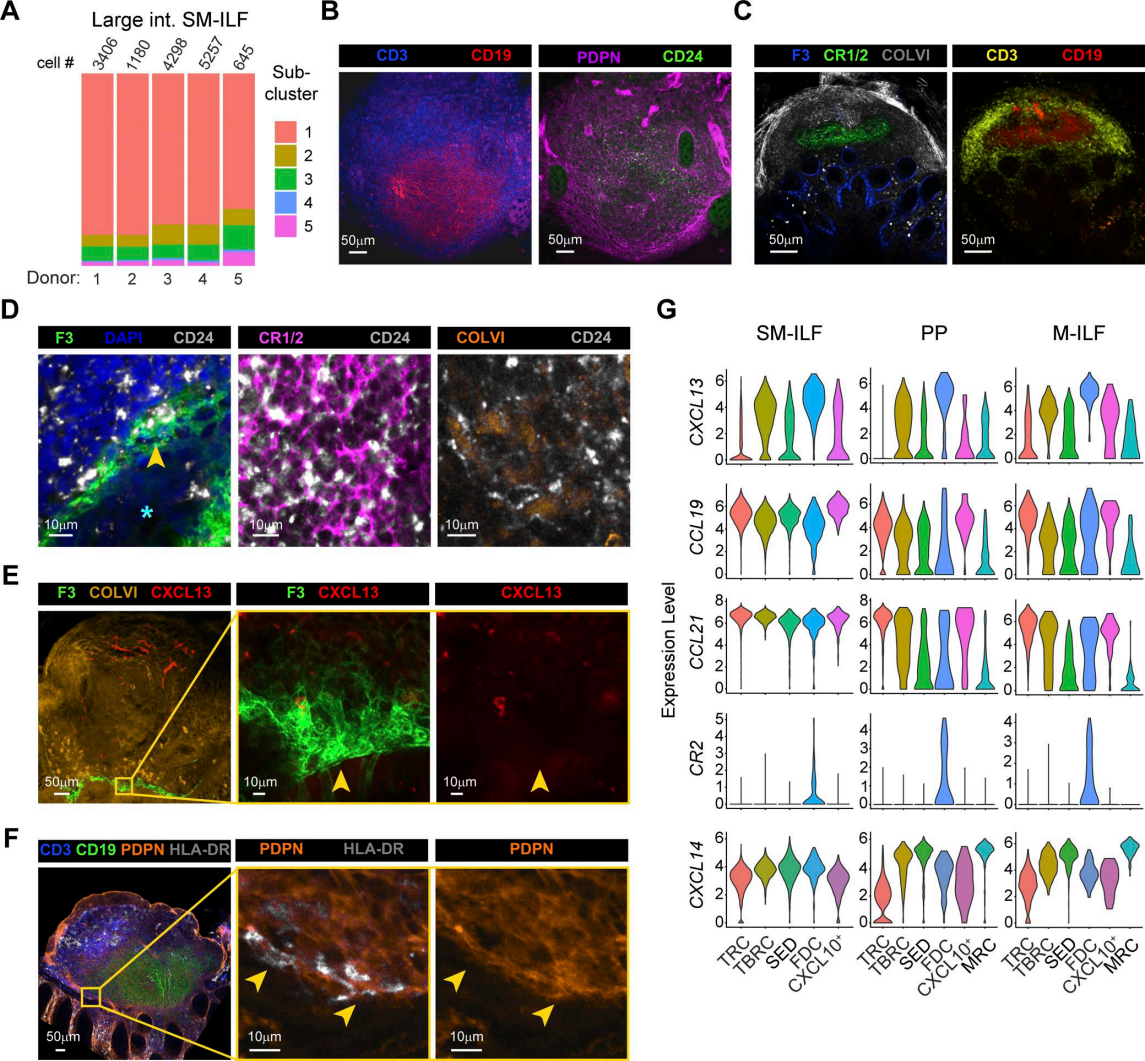
**FRC landscape of large intestinal SM-ILF and transcriptional differences to ileal GALT. (A)** Bar plots showing the abundance of the GALT FRC subclusters among all GALT FRC from large intestinal SM-ILF of five independently processed donors. **(B)** Confocal laser microscopy images showing the abundance of CD3^+^ T cells, CD19^+^ B cells (left panel), and CD24-expressing FRC among all PDPN^+^ FB (right panel) within large intestinal SM-ILF. **(C)** Confocal laser microscopy images showing the expression of CD24 on F3^+^/CR1/2^+^/COLVI^+^ FRC within large intestinal SM-ILF. **(D)** Confocal laser microscopy images showing the expression of CD24 on F3^+^/CR1/2^+^/COLVI^+^ FRC within large intestinal SM-ILF. Star marks the apical side of the epithelial layer facing the intestinal lumen. **(E)** Confocal laser microscopy images showing the expression of CXCL13 levels by FRC in the SED region. Yellow arrowheads indicate location of F3^+^ SED FRC. **(F)** Confocal laser microscopy images showing the expression of HLA-DR in large intestinal SM-ILF. Yellow arrowheads indicate location of PDPN^+^HLA-DR^+^ FRC. **(B–F)** Images show one representative image out of two or three replicates that were stained in independent experiments. **(G)** Violin plots showing the expression of the indicated genes by large intestinal SM-ILF, as well as ileal PP and M-ILF. Data are from five independently processed tissue donors (5× PP and SM-ILF, 3× M-ILF).

**Figure 5.**
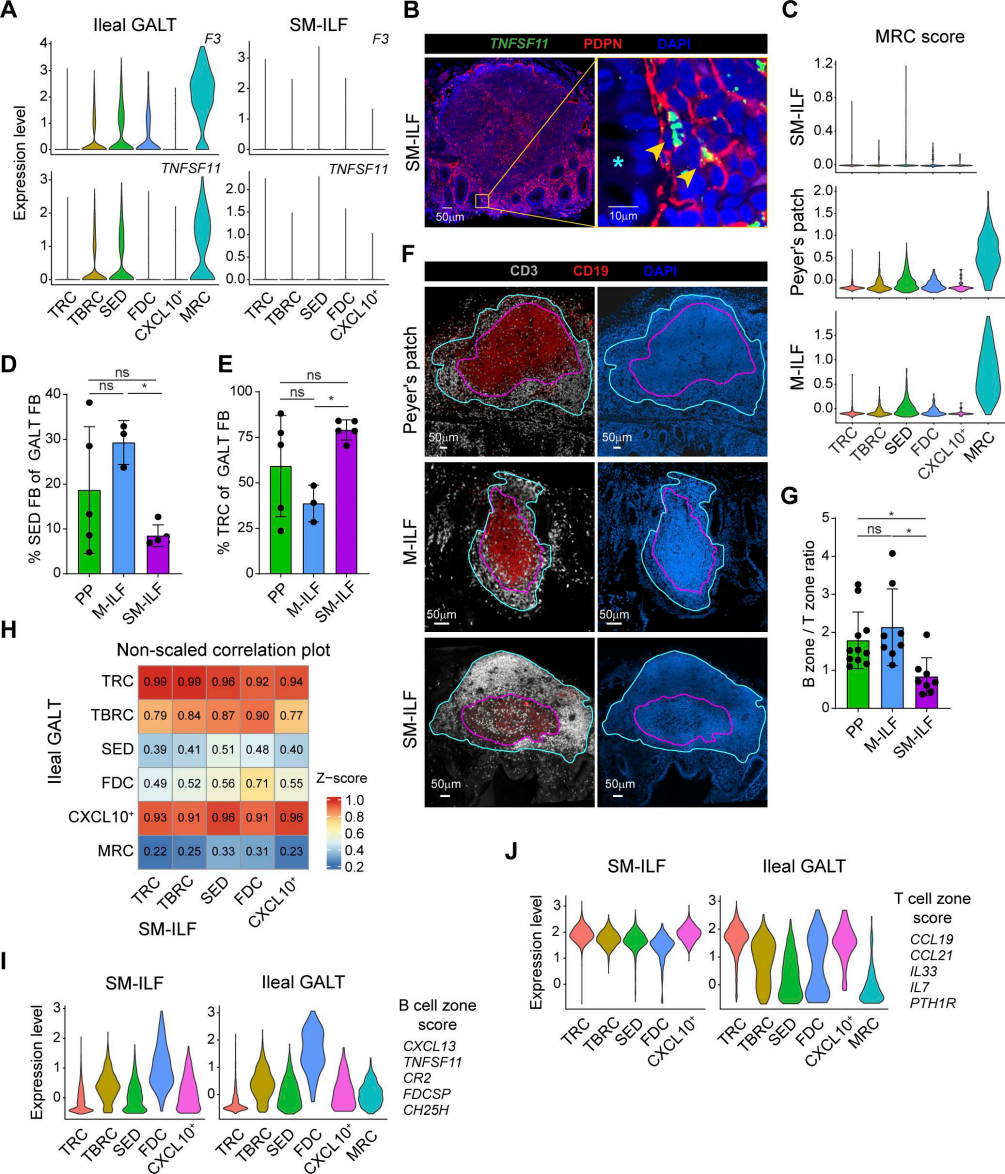
**Large and small intestinal GALT possess distinct FRC landscapes. (A)** Violin plots showing the expression levels of *F3* and *TNFSF11* by the indicated GALT FRC subclusters from ileal GALT (left plots) and large intestinal SM-ILF (right plots). **(B)** Confocal laser microscopy image of a large intestinal SM-ILF stained for PDPN and a *TNFSF11* RNA probe. Left plot: Overview image. Right plot: Zoomed-in region of the yellow square region of the left plot. Star marks the apical side of the epithelial layer facing the intestinal lumen, yellow arrowheads mark PDPN^+^*TNFSF11*^+^ FRC. Representative image from six replicates from different tissue donors (with two out of six showing *TNFSF11*^*+*^ cells). **(C)** Overlay of an MRC gene signature on PP, M-ILF, and SM-ILF FRC subclusters based on the top 10 DEG expressed by ileal GALT MRC. **(D–E)** Bar plots showing the abundance of SED-FRC (C) and TRC (D) among GALT FRC from indicated tissues. **(F)** Representative confocal laser microscopy image used for the quantification of T cell zone (purple) and B cell zone (turquoise) size within a GALT follicle. 8–11 follicles from different donors were analyzed per GALT type. **(G)** Bar plot showing the B and T cell zone ratio per follicle. Pooled data are from eight independent experiments. **(H)** Nonscaled comparison of expression profiles of the indicated GALT FRC subclusters in ileal GALT and large intestinal SM-ILF. Zero (blue) indicates no overlap of the gene signatures; one (red) indicates full overlap. **(I)** Overlay of a curated B cell zone score on large intestinal SM-ILF FRC (left plot) and ileal GALT (right plot) FRC subclusters. **(J)** Overlay of a curated T cell zone score on large intestinal SM-ILF (left plot) and ileal GALT (right plot) FRC subclusters. **(A, C–E, and H–J)** Data are from five independently processed donors (5× PP and SM-ILF, 3× M-ILF). **(C, D, and F)** Statistical significance between the samples was determined using a one-way ANOVA followed by Tukey’s multiple comparison test. Dots represent individual samples, bars represent means per group, and whiskers indicate SD. ns, not significant; *P < 0.05; differences with a P value <0.05 were considered statistically significant.

As our GALT FRC dataset also included 14,786 cells from large intestinal SM-ILF, we next assessed whether GALT FRC from SM-ILF differed from their ileal counterparts. DEG analysis between large intestinal SM-ILF and ileal GALT FRC identified 392 DEG (Fig. 4 A). We therefore analyzed FRC from SM-ILF separately, identifying five subclusters that were present in all SM-ILF samples (Fig. 4 B and Fig. S4 A) and all of which expressed *CD24* and *CLU* (Fig. 4 C). Analysis of top DEG and canonical FB markers together with immunohistochemical analysis was performed as for the ileal GALT FRC (Fig. 4, D and E; Fig. S4, B–F; and Table S3). Collectively, this analysis indicated that SM-ILF FRC subcluster 1 represented TRC, subcluster 2 represented TBRC, subcluster 3 represented SED-FRC, subcluster 4 represented FDC, and subcluster 5 represented *CXCL9*^*+*^*CXCL10*^+^ FRC (Fig. 4 F). Consistent with this, scaled correlation analysis, i.e., normalizing gene expression data by row, demonstrated that each of these clusters correlated most closely with their ileal GALT subcluster counterparts (Fig. 4 G). Despite these similarities, notable differences were observed between SM-ILF and ileal GALT FRC. Firstly, SM-ILF lacked a transcriptionally distinct MRC subcluster (Fig. 4 B and Table S3), and while SM-ILF FDC (cluster 4) and SED-FRC (cluster 3) expressed more *F3* and *TNFSF11* than other SM-ILF subclusters (Fig. 4 D), the expression of these genes was negligible compared with that observed in ileal GALT MRC (Fig. 5 A). Consistent with this finding, a minor population of *TNFSF11* expressing PDPN^+^ FB was observed in the SED region of only two of six SM-ILF analyzed (example in Fig. 5 B). Furthermore, when plotting an MRC gene score consisting of the top 10 DEG of the MRC cluster observed in ileal GALT, we found a low MRC signature across all large intestinal SM-ILF FRC subsets (Fig. 5 C). These observations are also consistent with previous findings that the SED regions of SM-ILF are small (Spencer et al., 2019). Indeed, while the proportion of SED-FRC among FRC in PP was highly variable (Fig. 5 C and Fig. S3 A), SM-ILF contained significantly lower proportions of SED-FRC than M-ILF (Fig. 5 D, Fig. S3 B, and Fig. S4 A). Secondly, while data from PP were again highly variable, FRC in SM-ILF contained significantly higher proportion of TRC compared with M-ILF (Fig. 4 B, Fig. S3 B, and Fig. S4 A). Consistent with this observation, the B zone-to-T zone ratio in SM-ILF was lower than in M-ILF (Fig. 5, F and G). Thirdly, when performing a comparison between ileal GALT and SM-ILF subclusters without scaling per row, to retain absolute gene expression differences between ileal GALT and SM-ILF, all SM-ILF subclusters correlated most closely with ileal GALT TRC and *CXCL9*^*+*^*CXCL10*^+^ FRC (Fig. 5 H), both of which displayed a strong T cell zone signature (Fig. 3 D). Indeed, while FDC and TBRC expressed the highest B cell signature score among FRC subclusters in both SM-ILF and ileal GALT (Fig. 5 I), and TRC and *CXCL9*^*+*^*CXCL10*^+^ FRC expressed the highest T cell zone signature score in ileal GALT (Fig. 5 J), all SM-ILF subclusters expressed a high T cell zone signature score (Fig. 5 J). Thus, SM-ILF FRC appear generally more poised toward T cell support.

### GALT FRC-like FB can be identified in intestinal biopsies and extraintestinal tissues

**Figure S5.**
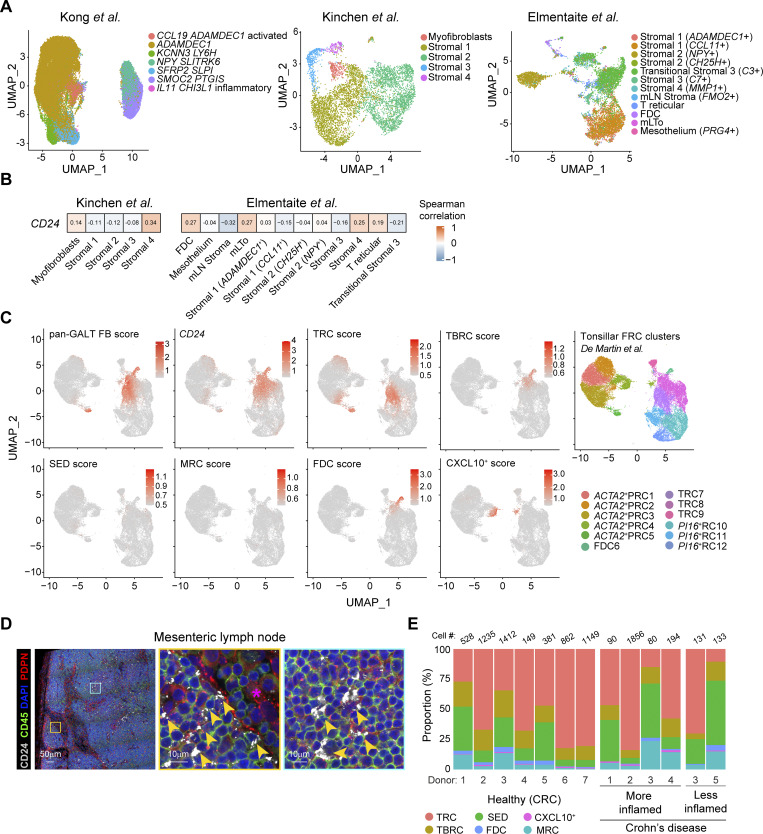
**Comparison of GALT** **FRC** **with other lymphoid tissue FB and with their counterparts during CD. (A)** UMAPs showing the intestinal stromal cell populations described by Kong et al. (2023), Kinchen et al. (2018), and Elmentaite et al. (2021) based on the original annotations. **(B)** Heatmap showing *CD24* expression by the FB clusters described in Kinchen et al. (2018) and Elmentaite et al. (2021). **(C)** UMAPs showing the expression of the indicated GALT FRC subset signatures (top 10 DEG per cluster) or *CD24* expression on the tonsillar FRC dataset published by De Martin et al., as well as the original tonsillar FRC clustering (De Martin et al., 2023). *ACTA2*^+^ PRC = α-smooth muscle actin–expressing PRC; *PI16*^+^ RC = peptidase inhibitor 16–expressing reticular cells. **(D)** Confocal laser microscopy images of mesenteric LN sections showing the expression of CD24 on PDPN^+^ FB and CD45^+^ immune cells. The boxes indicate magnified areas shown in the middle (yellow) and right panel (turquoise). Star marks a lymphatic sinus, yellow arrowheads indicate PDPN^+^CD24^+^ FRC. One image shown of three replicates from different donors that were stained in independent experiments. **(E)** Proportions of GALT FRC subsets in the indicated samples. The numbers above each bar indicate the number of GALT FRC per dataset.

**Figure 6.**
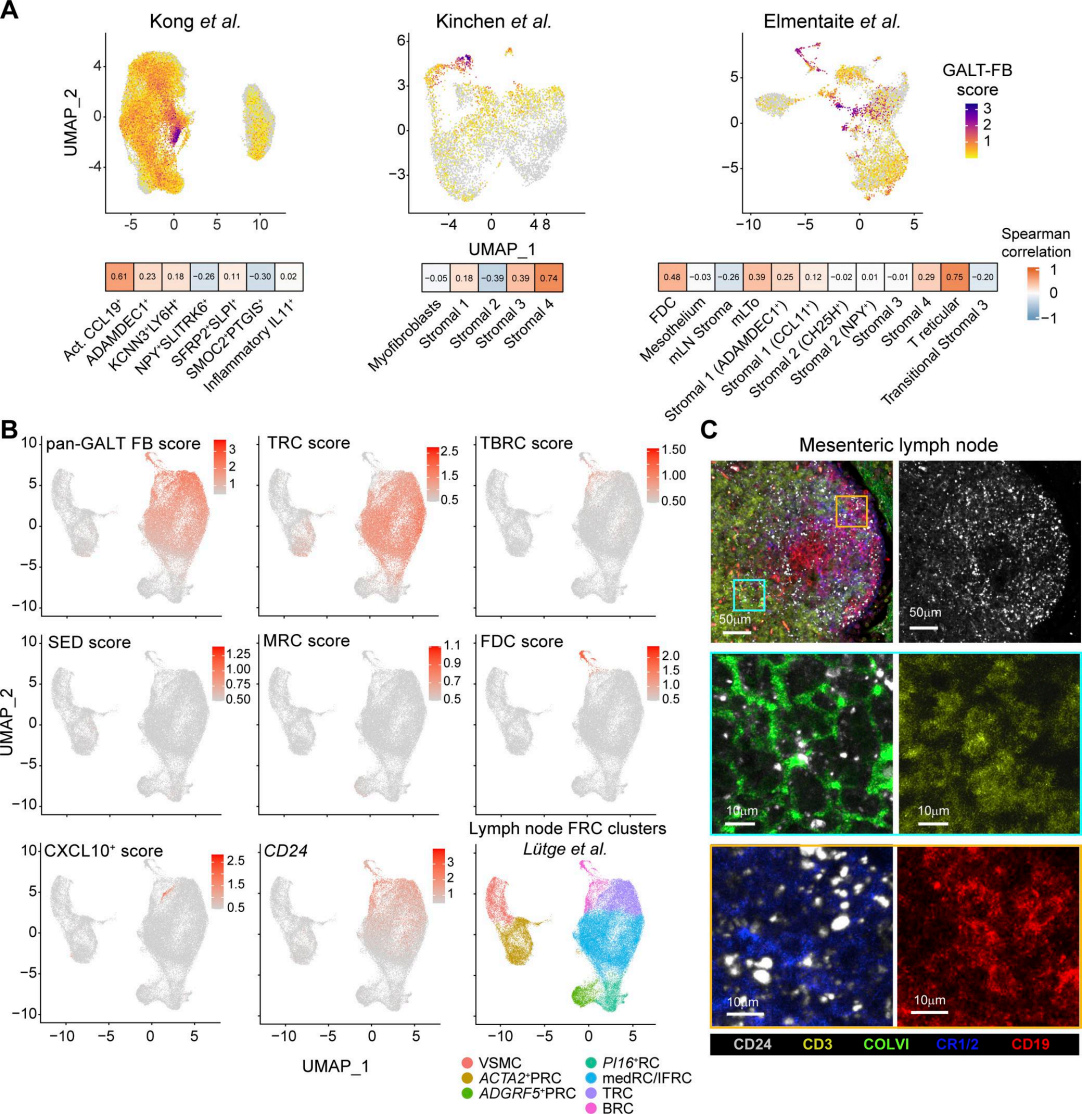
**GALT FRC-like FB can be detected in biopsy-based intestinal datasets and in human LN. (A)** Upper panels: UMAPs showing the GALT FRC signature score (top 10 DEG) overlaid on the intestinal FB datasets of Kong et al. (2023) (left panel), Kinchen et al. (2018) (middle panel), and Elmentaite et al. (2021) (right panel). Lower panels: Spearman’s correlation plots depicting the similarities between the GALT FRC signature identified in this study (mean is set to zero) by indicated FRC clusters. **(B)** UMAPs showing the expression of the indicated GALT FRC subset signatures (top 10 DEG per cluster) or *CD24* expression on the LN FRC dataset published by Lütge et al., as well as the original LN FRC clustering (Lütge et al., 2025). *ACTA2*^+^ PRC = α-smooth muscle actin–expressing PRC; *PI16*^+^ RC = peptidase inhibitor 16–expressing reticular cells. ADGRF5^+^ PRC = adhesion G protein–coupled receptor F5^+^ PRC; VSMC = vascular smooth muscle cells; medRC = medullary reticular cells; IFRC = interfollicular reticular cells. **(C)** Confocal laser microscopy images of a human mesenteric LN showing the expression of CD24 on COLVI^+^ and CR1/2^+^ FRC together with CD19^+^ B cells and CD3^+^ T cells. One representative image shown of replicates from two donors that were stained in independent experiments.

Having identified diverse FRC subsets within human GALT, we next assessed whether FB bearing similar transcriptional signatures could be identified in publicly available scRNA-seq datasets. Overlaying our pan-GALT FRC signature score, comprising the top 10 DEG of GALT FRC (Table S2), with intestinal FB datasets generated from pooled intestinal biopsies described by Kong et al. (2023), Kinchen et al. (2018), and Elmentaite et al. (2021) (Fig. S5 A), we found a high transcriptional overlap with a minor “activated *CCL19*^+^*ADAMDEC1*^+^” FB described by Kong et al., the proinflammatory *CCL19*^+^ “Stromal cluster 4” described by Kinchen et al., and the FDC and TRC-like clusters described by Elmentaite et al. (Fig. 6 A). In datasets that included the *CD24* gene, these cells also represented the FB subset that expressed the highest levels of *CD24* (Fig. S5 B). Given their rarity, it seems likely that such cells derive from *bona fide* ILF that were occasionally sampled in random biopsies of the mucosa. Notably, the GALT FRC signature score showed little overlap with previously described *IL11*^*+*^*OSMR*^*+*^*FAP*^+^ inflammatory FB (Friedrich et al., 2021; Kong et al., 2023; Martin et al., 2019; Smillie et al., 2019; West et al., 2017) included in the Kong et al. dataset (Fig. 6 A), indicating this IBD-associated FB subset is transcriptionally distinct from GALT FRC.

Overlaying our pan-GALT FRC signature score onto recently published stromal scRNA-seq datasets from human cervical LN (Lütge et al., 2025) and tonsils (De Martin et al., 2023) revealed that GALT FRC most closely aligned with bona fide lymphoid tissue FRC in both tissues (Fig. 6 B and Fig. S5 C). No overlap was observed with the more structural FB populations within these datasets, including perivascular reticular cells (PRC), *PI16*^+^ reticular cells, and smooth muscle cells (Fig. 6 B and Fig. S5 C). Cells with a GALT SED-FRC or F3^+^ FRC signature were absent from both lymphoid tissues (Fig. 6 B and Fig. S5 C). Further, while a few cells within the cervical LN TRC cluster displayed a GALT-like *CXCL9*^*+*^*CXCL10*^+^ FRC signature (Fig. 6 B), cells bearing such a signature in tonsils were restricted to an *ACTA2*-expressing PRC cluster (Fig. S5 C).

Notably, *CD24* expression overlapped with TRC and BRC/FDC in both tonsils and cervical LN (Fig. 6 B and Fig. S5 C), as well as with interfollicular and medullary FB within cervical LN (Fig. 6 B and Fig. S5 C), that also express *CCL19*, *CCL21*, and *CLU* (Lütge et al., 2025). Using confocal laser microscopy, we found that CD24 was expressed by PDPN-expressing FRC in immune cell–rich regions of human mesenteric LN, but not by PDPN^+^ cells within lymphatic sinuses (Fig. S5 D). This included both CR1/2^+^ FDC and ColVI^+^ FRC within T cell–rich areas (Fig. 6 C).

Collectively, these results indicate that human GALT, LN, and tonsils harbor partially overlapping, but also distinct, FRC clusters, and that CD24 serves as a marker of lymphoid tissue FRC across multiple human secondary lymphoid tissues.

### GALT FRC acquire an inflammation-associated transcriptional program during CD

**Figure 7.**
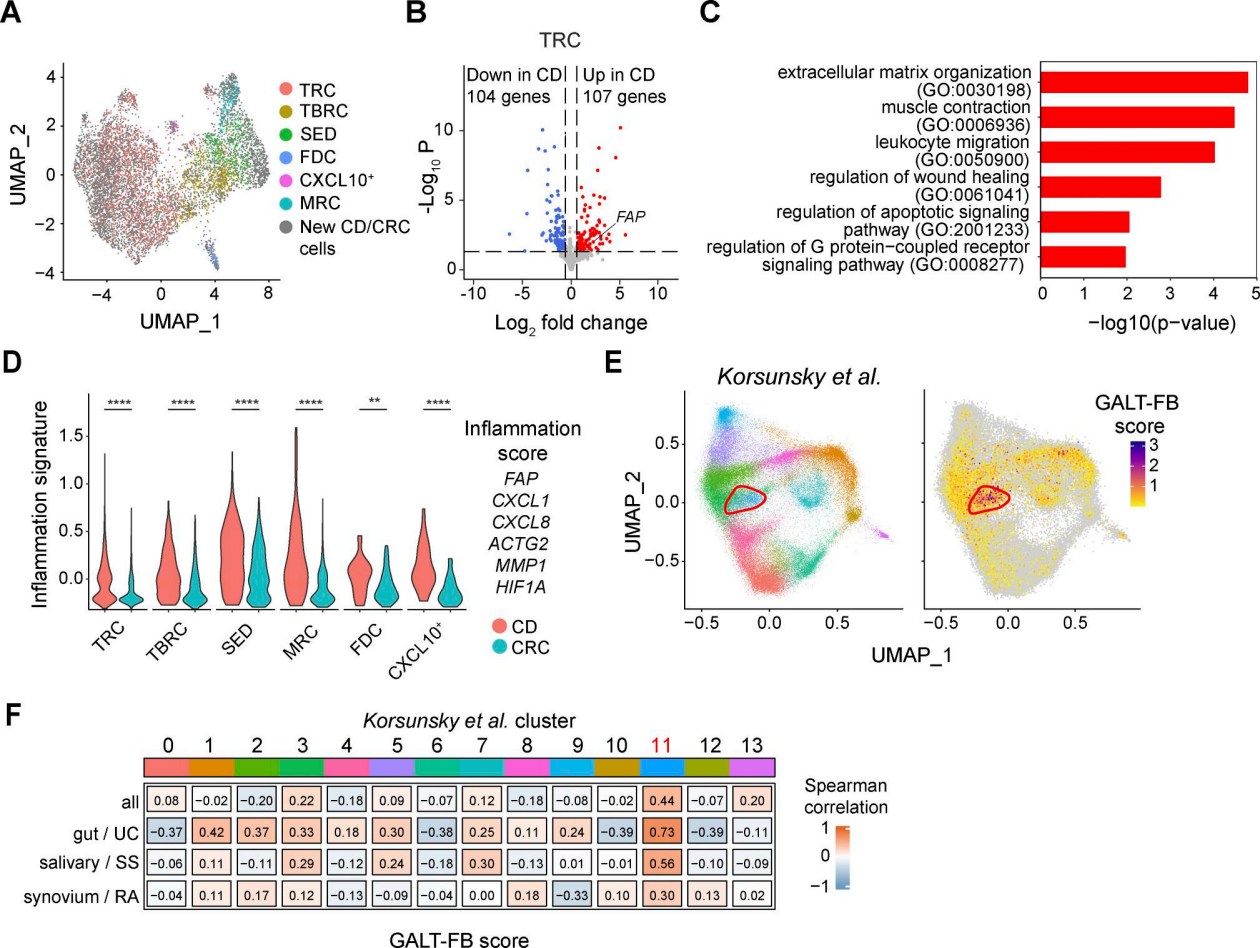
**Transcriptional profile of** **FRC** **from PP during chronic inflammatory disease. (A)** UMAP showing the bioinformatically isolated 8,200 GALT FRC derived from PP of seven independently processed donors with CRC and five with CD. The colored dots depict CRC donor-derived cells included in the ileal GALT dataset (Fig. 3); the gray cells depict the added PP-derived cells from two additional CRC and five CD donors. **(B)** Volcano plot showing the number of significantly up- and downregulated DEG among TRC from three inflamed donors with CD and seven non-inflamed donors with CRC, based on pseudobulk analysis using all samples with >30 TRC. Line marks *FAP*. **(C)** GO-term analysis based on the same samples than in A and B. **(D)** Overlay of an inflammation score composed of the indicated genes of GALT FRC from PP of donors with CD and CRC. Statistical significance between the samples was determined using a Wilcoxon rank sum test. **P < 0.01; ****P < 0.0001; differences with a P value <0.05 were considered statistically significant. **(E)** Original UMAP representation of the cross-tissue chronic disease stromal cell atlas by Korsunsky et al. (2022) (left plot) and overlay of the GALT FRC signature (top 10 DEG) on the dataset (right panel). Red circles indicate the location of the proinflammatory *CXCL10*^*+*^*CCL19*^*+*^ FRC cluster (cluster 11) identified by Korsunsky et al. (2022). **(F)** Spearman correlation plot showing the overlap between the GALT FRC cluster identified in this study and the indicated FRC clusters described by Korsunsky et al. (2022). Cluster 11 represents the disease activity-associated *CXCL10*^*+*^*CCL19*^*+*^ FB cluster (Korsunsky et al., 2022). UC, ulcerative colitis; SS, Sjögren’s syndrome; RA, rheumatoid arthritis.

To investigate whether the FRC landscape of human GALT is altered in CD, we performed scRNA-seq on six PP isolated from five donors with CD (four from more-inflamed and two from less-inflamed areas of ileum as judged macroscopically by a staff pathologist and confirmed by histological scoring [see Table S1]), as well as from two additional patients with CRC. Cells bearing a GALT FRC-like signature were identified, bioinformatically isolated, combined using Harmony-based integration with our prior GALT FRC datasets, and projected into a UMAP with the prior GALT FRC subset labels to identify the various FRC subsets (Fig. 7 A and Table S4). Every GALT FRC subset was represented in each of the CD PP samples albeit in varying proportions (Fig. S5 E). Given the prominent role of T cells in CD pathology (Neurath, 2019), the capacity of TRC to recruit T cells and modulate their functions (reviewed in De Martin et al. [2024]), and the fact that TRC represented the dominant GALT FRC subset in our datasets, we focused on comparing the transcriptional profile of TRC from inflamed PP of patients with CD with TRC from healthy PP from CRC samples. DEG analysis identified 211 DEG (Fig. 7 B and Table S4), and when mapping these DEG to biological processes (Gene Ontology [GO] terms, using GO.db version 3.18.0), we found genes upregulated in donors with CD to be involved in signaling pathways around leukocyte attraction, extracellular matrix organization, and contractile function (Fig. 7 C), reminiscent of changes observed in LP FB during IBD (Ke et al., 2024; Kong et al., 2023; Mukherjee et al., 2023). Among the CD-associated DEG was *FAP* (fibroblast activation protein) (Fig. 7 B), a gene previously associated with inflammatory FB in CD (Friedrich et al., 2021; Thomas et al., 2024) along with other known inflammation-related genes (Friedrich et al., 2021; Kong et al., 2023; Kong et al., 2025) including the chemokines *CXCL1* and *CXCL8*, the actin-encoding gene *ACTG2*, *MMP1* (matrix metalloprotease 1), and *HIF1A* (hypoxia-induced factor 1a) (Table S4). Cell numbers in other GALT FRC subsets were low and variable (Fig. S5 E), precluding detailed analysis; however, applying this inflammation gene signature revealed that changes seen in TRC were mirrored across the other GALT FRC subsets (Fig. 7 D).

Finally, to assess whether GALT FRC-like cells resemble any FB present in chronic inflammatory lesions of other inflammatory diseases, we mapped the GALT FRC signature score on a FB atlas derived from ulcerative colitis (intestine), rheumatoid arthritis (synovium), and Sjögren’s syndrome (salivary gland) lesions (Korsunsky et al., 2022) (Fig. 7 E). Among 14 transcriptionally distinct FB clusters identified across organs and conditions, the authors identified two conserved proinflammatory FB clusters, namely, *SPARC*^+^*COL3A1*^+^ perivascular FB (“cluster 4”) and *CXCL10*^+^*CCL19*^+^ immune-interacting FB (“cluster 11”) (Korsunsky et al., 2022). Cells with a GALT FRC-like signature aligned in each of these diseases with *CXCL10*^*+*^*CCL19*^*+*^ FB cluster 11 (Fig. 7 G), indicating that GALT FRC are transcriptionally similar to these cells.

## Discussion

Recent scRNA-seq analysis of intestinal biopsies has substantially advanced our understanding of FB diversity within the human intestinal LP, highlighting specialized populations of both interstitial and SE-FB (reviewed in Brügger and Basler [2023]). In the present study, we expand upon this knowledge by characterizing the FRC landscape of human GALT. We demonstrate that human GALT contain numerous transcriptionally and spatially restricted FRC subsets including specialized T cell zone–associated TRC, B cell zone–associated TBRC and FDC, *CXCL14*^*hi*^ FRC in the SED and interfollicular regions of PP, and variable amounts of *CXCL9*^*+*^*CXCL10*^+^-activated FRC. We also find notable differences in the FRC landscape of ileal and large intestinal GALT, with the former containing a clearer population of MRC in the SED and the latter expressing a more T zone–like profile. Lastly, we found that PP of patients with CD contain similar GALT FRC subsets and comparison with previously published datasets suggested that FRC-like cells transcriptionally similar to our broad GALT FRC cluster exist in lesions of a range of chronic inflammatory diseases.

Our study identified the GPI-anchored sialoglycoprotein CD24 (Altevogt et al., 2021) as a novel marker that distinguished GALT FRC from other intestinal FB populations. While the functional relevance of CD24 on GALT FRC remains to be determined, the selective expression of CD24 by GALT FRC was also observed in FB bearing a FRC-like signature profile in intestinal biopsies (Elmentaite et al., 2021; Kinchen et al., 2018), mesenteric LN, cervical LN (Lütge et al., 2025), and human tonsils (De Martin et al., 2023). Thus, CD24 may be a useful marker to isolate and study FRC from tissue digestions where direct isolation of lymphoid tissues is not possible. Within the population of GALT FRC, we identified FB subsets that resembled FRC subsets previously described in murine secondary lymphoid organs and PP (Pikor et al., 2020; Prados et al., 2021; Rodda et al., 2018). Notably, such diversity in GALT FRC subsets was also observed in human ILF, contrasting markedly from murine ILF, which have been reported to contain a relatively homogeneous population of *CCL19*^*+*^*CXCL13*^*+*^ FB (Cheng et al., 2022; Tsuji et al., 2008). Consistent with this, lymphocyte subset composition is far more diverse in human compared with murine ILF (Fenton et al., 2020; Junker et al., 2009; Pabst et al., 2005), with murine ILF suggested to primarily contribute to T cell–independent B cell responses (Tsuji et al., 2008).

We observed several differences between the FRC landscape of ileal and large intestinal ILF with the former containing more transcriptionally defined clusters and a larger fraction of SED and follicle-associated FRC, and the latter expressing a transcriptional profile more focused toward T cell recruitment and support. While the underlying reason for these differences remains to be determined, ileal M-ILF have a larger FAE and SED than large intestinal SM-ILF (Fenton et al., 2020; Jørgensen et al., 2021; Spencer et al., 2019). We thus speculate that these differences in FRC landscape may be driven by the relative exposure of these structures to luminal antigen, with greater sampling in ileal M-ILF fueling enhanced B cell follicle formation and FRC specification. Consistent with this possibility, large intestinal SM-ILF contain greater proportions of naïve CD4^+^ and CD8^+^ T cells and reduced proportions of GC B cells compared with ileal M-ILF (Fenton et al., 2020; Mörbe et al., 2021; Spencer et al., 2019). Whether differences in the FRC landscape of ileal M-ILF and large intestinal SM-ILF impact on the type of adaptive immune responses generated at these locations remains unclear.

The LP of patients with IBD contains a *de novo* population of inflammation-associated *FAP*^+^*IL11*^+^ FB (Friedrich et al., 2021; Smillie et al., 2019; Thomas et al., 2024; Zhang et al., 2024) that are thought to contribute to inflammatory monocyte recruitment and fibrosis (Ke et al., 2024; Zhang et al., 2024). Here, we found FRC diversity in PP of patients with CD to be like that of healthy PP, indicating that CD does not fundamentally alter FRC heterogeneity in GALT. Nevertheless, PP TRC from patients with CD upregulated *FAP* and genes in signaling pathways associated with extracellular matrix organization, leukocyte recruitment, and cell contraction, which was also recapitulated in the other GALT FRC subsets. Since these pathways have also been associated with *FAP*^+^*IL11*^+^ FB in the LP (Friedrich et al., 2021; Ke et al., 2024; Kong et al., 2023; Zhang et al., 2024) and with *FAP*^*+*^ FB that accumulate in rheumatoid arthritis and tumor microenvironments (Croft et al., 2019; Gao et al., 2024), we hypothesize that such changes in TRC are, at least in part, a general response of FB to chronic inflammation.

Several studies have demonstrated that FRC-like FB, reminiscent of the GALT FRC described here, are expanded in active IBD (Elmentaite et al., 2021; Kinchen et al., 2018; Thomas et al., 2024). Whether this increase is due to an expansion of existing GALT (Jørgensen et al., 2021), *de novo* formation of lymphoid aggregates (Sura et al., 2011; Thomas et al., 2024), or that diffusely distributed LP FB take on a GALT FRC-like phenotype in IBD, is yet to be determined. Further, while GALT FRC cells are essential in adaptive immune activation and regulation, whether such an increase in GALT FRC impacts disease progression remains unclear. In this regard, our observation that FB with a GALT FRC-like transcriptional profile are present in inflammatory lesions of extraintestinal inflammatory diseases and correlate with inflammatory status (Korsunsky et al., 2022) indicates they may have a proinflammatory role in disease.

In conclusion, we demonstrate that human GALT contain numerous transcriptionally and spatially distinct FRC subsets whose composition and transcription differ between different types of GALT and in PP from patients with CD. While the impact such differences have on adaptive immune priming and differentiation requires further study, our results provide an important framework for understanding GALT diversity and function.

### Limitations of this study

The isolation and analysis of human GALT require processing of intestinal surgical resections. We cannot exclude that tissue processing caused death of specific cell types resulting in their potential underrepresentation, as, for example, previously noted for FDC (Heesters et al., 2021). Healthy GALT tissue was isolated from surgical resections from patients with CRC, and it remains possible that GALT homeostasis is impacted by the presence of distant tumors. Further, since age impacts on lymphoid tissue organization and function (Foster et al., 2025; Senda et al., 2019), GALT FRC subset proportions and their transcriptional profiles, as well as the zonal organization of GALT, may differ in younger cohorts of individuals, which should also be considered when comparing tissues from older patients with CRC to younger patients with CD (Table S1). In addition, scRNA-seq–based cell profiles may not be fully reflected on a protein level due to posttranscriptional regulation. Integrating transcriptomics and proteomics data in future studies will be required to overcome this limitation. Finally, our requirement for surgical material restricted our analysis to a limited subgroup of patients with CD (Verstockt et al., 2022) and the low numbers of obtained inflamed PP, as well as the low cell yields, limited our abilities to thoroughly analyze the impact CD on all GALT FRC subsets. Larger and more diverse cohorts of patients will be required to fully understand GALT FRC alterations and functions in IBD.

## Materials and methods

### Intestinal tissues

Intestinal surgical tissue was obtained from patients with CRC or CD after informed consent and according to the principles of the Declaration of Helsinki and local ethical guidelines of the Scientific Ethics Committee of the Copenhagen Capital Region (Institutional Review Board approval numbers H-3-2012-118 and H-20054066). Tissues from both male and female donors were used, and donors older than 85 years and smokers (patients with CD) were excluded. For patients with CRC, proximal large intestine and ileal surgical samples were obtained >10 cm distant to the cancer lesion with ileal tissues taken <20 cm distant from the ileocecal junction and only samples from treatment-naïve donors were considered. For patients with CD, inflamed and non-inflamed regions (as macroscopically determined by an on-site pathologist) of distal ileum were obtained <60 cm from the ileocecal junction. The inflammatory status of each CD sample was determined using a modified D’Haens score (D’Haens et al., 1998). Briefly, one to two hematoxylin-and-eosin–stained sections were prepared per sample and scored by a qualified pathologist for the presence of epithelial damage, architectural changes, infiltration of mononuclear cells and polymorphonuclear cells in the LP, infiltration of polymorphonuclear cells in the epithelium, presence of erosion and/or ulcers, and presence of granuloma (D’Haens et al., 1998). All patient information can be found in Table S1.

### Generation of single-cell suspensions from intestinal compartments

Surgically removed tissues were taken up in R5 medium (RPMI-1640 medium [Gibco] containing FBS [5%] and 100× penicillin–streptomycin–glutamine [1%, Thermo Fisher Scientific]) and processed within 2 h of surgery. The different intestinal immune sites were isolated as previously described (Fenton et al., 2020; Jørgensen et al., 2021). Briefly, the muscularis externa was removed and mucus dissolved using dithiothreitol (4 mM, Thermo Fisher Scientific) in R5 medium. The SM distant to the mucosa was carefully removed using a pair of scissors; the remaining SM was separated from the mucosa using two pairs of curved forceps. To visualize SM-ILF, the SM was incubated for 2 min in methylene blue (0.1%, Sigma-Aldrich) in PBS (Thermo Fisher Scientific), and washed with PBS and PBS containing EDTA (5 mM, Thermo Fisher Scientific). Methylene blue–stained SM-ILF were excised, and GALT-free SM was collected separately in R5 medium. The separated ileal and large intestinal mucosa layers (without a PP) were incubated with HBSS/EDTA buffer (Ca^2+^- and Mg^2+^-free HBSS [Thermo Fisher Scientific] containing EDTA [5 mM]) at 37°C for 15 min per wash on an orbital shaker. HBSS/EDTA washes were repeated three to four times to remove the epithelial layer. Visible M-ILF within the washed mucosa layer were isolated under a stereomicroscope using a scalpel. PP were already visible within ileal tissues after the removal of the muscularis externa, excised, washed in HBSS/EDTA buffer, and stored in R5. After separation of GALT, LP, and SM, the separate fractions were enzymatically digested using R5 medium containing collagenase D (1.6 mg/ml, Sigma-Aldrich) and DNase I (0.15 mg/ml, Sigma-Aldrich) for 45 min at 37°C and constant agitation. The resulting cell suspensions were filtered through a mesh filter (100 µm), washed in R5 medium, and either processed immediately for cell sorting, flow cytometry, and scRNA-seq, or frozen for later use in flow cytometry.

### Flow cytometry analysis and cell sorting

To avoid unspecific binding of staining antibodies, single-cell suspensions were blocked in PBS containing FBS (2%, Sigma-Aldrich) and mouse serum (3%, Sigma-Aldrich) for 20 min and subsequently stained with fluorescently labeled antibodies (Table S5) diluted in brilliant stain buffer (BD Biosciences). Dead or dying cells were identified using 7-AAD, SYTOX Green, or LIVE/DEAD Fixable Far Red Dead Cell Stain (all Thermo Fisher Scientific) and excluded from subsequent analysis. Stained cells were washed twice in PBS containing FBS (2%) and analyzed using an Aria II cell sorter or an LSRFortessa II flow cytometer (BD Biosciences). For scRNA-seq, either living CD235ab^−^EpCAM^−^CD45^−^CD38^low^CD31^−^ cells (donors with CRC 1–3, donor with CD 4; Fig. S1) or all living CD235ab^−^EpCAM^−^CD45^−^ cells (donors with CRC 4–7, donors with CD 1–3, 5) were sorted. Postacquisition analysis was performed using FlowJo software version 10 (BD Biosciences).

### Singe-cell transcriptomics library generation and sequencing

After sorting, cells were spun down for 5 min at 400 *g* at 4°C, resuspended in PBS containing BSA (2%, Sigma-Aldrich), and subjected to scRNA-seq using the Chromium Single Cell 3′ Kit version 3 or 5′ Kit version 2 (both 10x Genomics), following the manufacturer’s instructions. Libraries were checked for quality and size using KAPA Library Quantification Kit for Illumina Platforms (Kapa Biosystems) and a High Sensitivity DNA chip with 2100 Bioanalyzer (Agilent). Multiplexed libraries were pooled and sequenced using a NextSeq 500/550 (150 cycles) at the Center of Excellence for Fluorescent Bioanalytics (KFB, University of Regensburg, Regensburg, Germany) or a NovaSeq 6000 (200 cycles) at the SNP&SEQ Technology Platform. A targeted sequencing depth of 30,000 reads per cell was chosen for gene expression libraries to ensure a sufficient sequencing saturation.

### Processing and analysis of own scRNA-seq datasets

scRNA-seq data were aligned to the hg38 reference genome using CellRanger (versions 2.2.0, 4.3.2, or 8.0.0); filtered feature-barcode matrices were read into R (version 4.3.2) as Seurat objects (version 4.4.0) and analyzed in accordance with current best practices (Hao et al., 2021; Heumos et al., 2023). Doublets, and low-quality and stressed cells were identified and removed on the basis of abnormally high or low levels of expressed genes and the relative abundance of mitochondrial transcripts. Data were normalized, variable genes were identified, and gene expression was scaled using Seurat, regressing out cell-cycle scores and mitochondrial and ribosomal content. Principal component analysis was performed on the normalized data, along with Harmony integration (Korsunsky et al., 2019) using sequencing batch, intestinal sample site, and patient ID as covariates. The Harmony-corrected embeddings were used for UMAP dimensionality reduction and Louvain algorithm–based clustering. Clusters were identified based upon DEG obtained using the Seurat “FindAllMarkers” function with default parameters and comparisons with previous publications (Elmentaite et al., 2021; Hickey et al., 2023; Kinchen et al., 2018). Contaminating non-FB subsets (key signature markers indicated in Fig. S1) were removed, and remaining FB populations were subsequently reanalyzed. FB clustering was refined in a semi-supervised manner with aggregation of transcriptionally similar populations differing in markers indicative of cell state such as activation. GALT FRC populations were annotated based upon DEG identified using a Wilcoxon rank sum test considering only markers expressed in >10% of cells in a cluster, and known murine FRC subset signature genes (Lütge et al., 2021; Prados et al., 2021; Rodda et al., 2018) with clusters identified as contaminating non-FRC cells removed. Pseudobulk DEG testing was performed on aggregated raw read counts for each sample using DESeq2 (version 1.42.1 [Love et al., 2014]) with sequencing batch, intestinal sample site, and patient ID as covariates. Log fold-change shrinkage was performed with apeglm (Zhu et al., 2019); significance was defined by an adjusted (Bonferroni-corrected) P value <0.05 and a log_2_ fold change less than −0.58 or >0.58. For the pseudobulk analysis–based comparison of TRC from patients with CD or CRC, only samples with ≥30 TRC were included to increase analysis robustness, and the GO analyses were based on the GO knowledge base (Ashburner et al., 2000; Aleksander et al., 2023). The statistical comparison between the GALT FB from donors with CD and CRC was performed on a single-cell basis using a Wilcoxon rank sum test.

### Processing and analysis of published scRNA-seq datasets

To compare our GALT FRC with other FB, we processed publicly available datasets of tonsillar (De Martin et al., 2023), LN (Lütge et al., 2025), intestinal (Kinchen et al., 2018; Kong et al., 2023; Elmentaite et al., 2021), and cross-tissue (Korsunsky et al., 2022) FB. Fetal tissue samples and samples from intestinal regions not included in our own data were removed, as well as low-quality cells, doublets, and stressed cells based on relative abundance of mitochondrial genes. Each sample in the datasets was processed separately, in the same manner applied to our own datasets as outlined previously. FB subsets were annotated using author-provided metadata whenever possible, or, for Kinchen et al. (2018), through identification of cluster markers. Seurat module scores were generated using the top 10 DEG based upon significance from each author annotated cluster and our GALT FRC (adjusted P value <0.05, Tables S2 and S3). Correlations between author annotated and GALT FRC scores were subsequently calculated for each respective cluster.

### Histological analysis

Tissue was incubated in 4% paraformaldehyde (Sigma-Aldrich) for 6–12 h for fixation and washed in wash/stain buffer (PBS containing FBS [5%], Triton X-100 [0.2%], all from Sigma-Aldrich). For sectioning, fixed tissues were embedded in low melting agarose (4%, Invitrogen) and sectioned with a swinging blade microtome (VT1200S; Leica). 50–100 μm sections were blocked in wash/stain buffer containing mouse serum (3%), and incubated with directly labeled or unlabeled primary antibodies (Table S5) in wash/stain buffer for 12–36 h at 4°C and, when relevant, with fluorescent secondary antibodies for 12 h. Nuclei were stained with 4′,6-diamidino-2-phenylindole (300 nM, DAPI, Thermo Fisher Scientific). Finally, tissues were washed in wash/stain buffer for 4–6 h and mounted on glass slides with ProLong Gold Glass antifade solution (Thermo Fisher Scientific). For combined antibody and fluorescence *in situ* hybridization (FISH) analysis, 3- to 5-μm sections from formalin-fixed paraffin-embedded tissue blocks were mounted on glass slides. Sections were incubated for 12–16 h at 4°C with RNAscope Co-detection Antibody Diluent containing unlabeled antibody against PDPN (Table S5) (R&D), washed in PBS containing Tween-20 (0.1%), permeabilized, and hybridized with the indicated probes (Table S5). Probes were detected using TSA Vivid fluorophores, and the antibody against PDPN was detected with an NL557-labeled anti-goat secondary antibody (R&D) for 30 min at room temperature. Nuclei were counterstained using DAPI (300 nM) and stained samples washed once with water and prepared for imaging as above. Images were acquired using an LSM710, LSM900, or LSM980 confocal laser microscope (Zeiss) with an EC Plan-Neofluar 40×/1.3 objective or an LD Plan-Neofluar 20×/0.4 objective and Zen Black 2012 software (Zeiss). Images were exported using Zen version 2.3 and processed using Imaris version 8 or Imaris Viewer version 10 (Oxford Instruments).

#### Determination of T and B zone size

One tissue section per GALT type and donor was selected, the central B cell zone was identified by a high density of CD19^+^ B cells, and the marginal T cell zone by a high density of CD3^+^ T cells, and the DAPI channel was considered for the identification of cell dense follicles and organ margins. If the center and margins of the follicle were not visible in a single plane, a limited z-stack was acquired, and images were projected into one layer using the maximum intensity projection tool. The areas of each zone were determined using the draw spine contour tool in the Zeiss Zen software, and statistical analysis was performed using Prism 10 (GraphPad).

### Online supplemental material

Fig. S1 shows the sorting strategy for scRNA-seq experiments and downstream analysis of scRNA-seq FB data and other contaminating cells in the dataset. Fig. S2 shows flow cytometry analysis of MHCII expression on intestinal FB subsets and immunohistochemical analysis demonstrating the location of the four major FB subsets in human large intestine and SM-ILF. Figs. S3 and S4 provide additional bioinformatics and histological analyses of ileal GALT FRC (Fig. S3) and large intestinal SM-ILF–derived GALT FRC (Fig. S4). Fig. S5 presents more detailed analyses of scRNA-seq FB data from the previously published datasets shown in Figs. 6 and 7, along with additional histological analyses of human LN sections and a summary of cell numbers included in the CD datasets. Table S1 shows the tissue donor information. Table S2 contains the DEG lists for the four broad intestinal FB clusters. Table S3 contains the DEG lists for the GALT FRC subclusters in ileal GALT and large intestinal SM-ILF. Table S4 contains the DEG lists and GO-term analyses based on additional GALT FRC datasets from donors with CD and control donors with CRC (shown in Figs. 7 and S4). Table S5 contains the antibodies, staining reagents, and RNAscope probes used for flow cytometry and/or histology.

## Supplementary Material

Table S1 shows the tissue donor information.

Table S2 contains the DEG lists for the four broad intestinal FB clusters.

Table S3 contains the DEG lists for the GALT FRC subclusters in ileal GALT and large intestinal SM-ILF.

Table S4 contains the DEG lists and GO-term analyses based on additional GALT FRC datasets from donors with CD and control donors with CRC (shown in Figs. 7 and S4).

Table S5 contains the antibodies, staining reagents, and RNAscope probes used for flow cytometry and/or histology.

## Data Availability

The scRNA-seq datasets and metadata generated in this study can be accessed via the BioStudies database (https://www.ebi.ac.uk/biostudies/) and are available under the accession code S-BSST2281. To protect patient confidentiality in accordance with the EU GDPR and Danish data protection laws, scRNA-seq data are provided in an anonymized form. Specifically, data are available as read count matrices and as *FASTQ* files, in which patient-specific genetic sequence variants were removed using the BAMboozle pipeline (Ziegenhain and Sandberg, 2021), which reverts patient-specific single nucleotide polymorphisms to the corresponding sequence of the human reference genome. Anonymized flow cytometry and histology raw data are available upon reasonable request to the corresponding authors.
